# A metabolic core model elucidates how enhanced utilization of glucose and glutamine, with enhanced glutamine-dependent lactate production, promotes cancer cell growth: The WarburQ effect

**DOI:** 10.1371/journal.pcbi.1005758

**Published:** 2017-09-28

**Authors:** Chiara Damiani, Riccardo Colombo, Daniela Gaglio, Fabrizia Mastroianni, Dario Pescini, Hans Victor Westerhoff, Giancarlo Mauri, Marco Vanoni, Lilia Alberghina

**Affiliations:** 1 SYSBIO Centre of Systems Biology, Milano, Italy; 2 Dept of Informatics, Systems and Communication, University Milano-Bicocca, Milano, Italy; 3 Institute of Molecular Bioimaging and Physiology, CNR, Segrate, Milan, Italy; 4 Dept of Biotechnology and Biosciences, University Milano-Bicocca, Milano, Italy; 5 Dept of Statistics and Quantitative Methods, University Milano-Bicocca, Milano, Italy; 6 Dept of Molecular Cell Physiology, Faculty of Earth and Life Sciences, VU University, Amsterdam, The Netherlands; 7 Manchester Centre for Integrative Systems Biology, School of Chemical Engineering and Analytical Science, University of Manchester, Manchester, United Kingdom; 8 Swammerdam Institute for Life Sciences, Faculty of Science, University of Amsterdam, Amsterdam, The Netherlands; University of Michigan, UNITED STATES

## Abstract

Cancer cells share several metabolic traits, including aerobic production of lactate from glucose (Warburg effect), extensive glutamine utilization and impaired mitochondrial electron flow. It is still unclear how these metabolic rearrangements, which may involve different molecular events in different cells, contribute to a selective advantage for cancer cell proliferation. To ascertain which metabolic pathways are used to convert glucose and glutamine to balanced energy and biomass production, we performed systematic constraint-based simulations of a model of human central metabolism. Sampling of the feasible flux space allowed us to obtain a large number of randomly mutated cells simulated at different glutamine and glucose uptake rates. We observed that, in the limited subset of proliferating cells, most displayed fermentation of glucose to lactate in the presence of oxygen. At high utilization rates of glutamine, oxidative utilization of glucose was decreased, while the production of lactate from glutamine was enhanced. This emergent phenotype was observed only when the available carbon exceeded the amount that could be fully oxidized by the available oxygen. Under the latter conditions, standard Flux Balance Analysis indicated that: this metabolic pattern is optimal to maximize biomass and ATP production; it requires the activity of a branched TCA cycle, in which glutamine-dependent reductive carboxylation cooperates to the production of lipids and proteins; it is sustained by a variety of redox-controlled metabolic reactions. In a *K-ras* transformed cell line we experimentally assessed glutamine-induced metabolic changes. We validated computational results through an extension of Flux Balance Analysis that allows prediction of metabolite variations. Taken together these findings offer new understanding of the logic of the metabolic reprogramming that underlies cancer cell growth.

## Author summary

Hallmarks describing common key events in initiation, maintenance and progression of cancer have been identified. One hallmark deals with rewiring of metabolic reactions required to sustain enhanced cell proliferation. The availability of molecular, mechanistic models of cancer hallmarks will mightily improve optimized personal treatment and new drug discovery. Metabolism is the only hallmark for which it is currently possible to derive large scale mathematical models, which have predictive ability. In this paper, we exploit a constraint-based model of the core metabolism required for biomass conversion of the most relevant nutrients—glucose and glutamine—to clarify the logic of control of cancer metabolism. We newly report that, when available oxygen is not sufficient to fully oxidize available glucose and glutamine carbons–a situation compatible with that observed under normal oxygen conditions in human and in cancer cells growing *in vitro*—utilization of glutamine by reductive carboxylation and conversion of glucose and glutamine to lactate confer advantage for biomass production. Redox homeostasis can be maintained through the use of different alternative pathways. In conclusion, this paper offers a logic interpretation to the link between metabolic rewiring and enhanced proliferation, which may offer new approaches to targeted drug discovery and utilization.

## Introduction

Cancer is a network disease [1] resulting from a variable combination of genetic and epigenetic alterations and selection of selfish cells, which maximize their fitness at the expense of the organism [2]. Despite the high morphological, genetic and molecular heterogeneity [3], cancer cells share a restricted number of essential alterations in cell physiology, better known as hallmarks [4]. A reorganization of the hallmarks [5] identifies in the enhanced, unrestricted cell growth the basic property of cancer cells.

This property requires an extensive reorganization of metabolic fluxes [6–9], characterized by an enhancement in aerobic glycolysis—the well-known Warburg effect [10]—and by the utilization of glutamine as a source of both carbon and nitrogen to support cellular biosyntheses. While the involvement of glucose in cancer cell metabolism has been widely studied, its integration with the utilization of glutamine through a reductive carboxylation pathway [11–18] leaves many aspects unresolved, given that the two pathways appear to be decoupled [19], but somehow coordinated [20]. These perturbations in key metabolic pathways not only propel cells towards malignancy, but also drive changes in the tissue microenvironment–ultimately helping cells to break through the physical constraints of their surrounding stroma and to evade immune recognition [21]. A deeper understanding on how cancer metabolic rewiring (CMR) drives initiation, maintenance and progression of the disease would thus be of considerable interest.

In order to integrate sets of experimental data and to formulate new testable hypotheses, complex biological systems–such as human metabolism that involves thousands of metabolites and reactions—need to be formally described by mathematical models. In this regard, constraint-based modeling and in particular Flux Balance Analysis (FBA), which is based on the definition and manipulation of stoichiometric matrices and was originally developed for the optimization of microbial strains, is rapidly gaining popularity within life sciences [22]. FBA allows to identify a phenotype that maximizes a certain objective (typically growth) among all the possible flux patterns compatible with the steady state assumption. In biological terms, these flux patterns may result from the expression and catalytic competence of the corresponding enzymes according to substrate availability, or, to use our terminology, from different wirings.

FBA may be applied to genome-wide metabolic networks [23, 24], which include the stoichiometry of the vast majority of chemical reactions that are catalyzed by enzymes encoded by the human genome. These maps have been effectively exploited as a scaffold for ‘omic’ data integration, proving able to identify essential genes and reporter metabolites in metabolic disorders and cancer [24–26]. In data-driven FBA studies of cancer metabolism [27, 28], aerobic glycolysis has been typically imposed on the network. However, if we want to derive the design principles that control metabolic rewiring in cancer cells and link them to enhanced proliferation, we need to let CMR emerge from the simulated boundary conditions.

Being highly detailed, genome-wide models require many assumptions on *in-vivo* nutrient utilization rates. Moreover, when the aim is to investigate the plethora of admissible wirings, rather than to determine optimality, their computation becomes demanding and may give origin to a combinatorial explosion of different flux patterns, which fuels mere details while masking the major regulatory connections. Manually curated core models including hundreds of metabolites, selected for the specific purpose of the analysis, help to overcome the problems above [29, 30]. We therefore expect them to increase the knowledge on the system and generate novel, experimentally testable predictions.

In this study, we aim to identify the regulatory principles that link the utilization of glucose and glutamine in originating the Warburg effect, and ultimately in driving cancer cell growth. We manually reconstructed a core stoichiometric model designed to evaluate the contribution of glucose and glutamine to enhanced growth, hence the name ENGRO1 (ENhanced GROwth). By exploring the space of admissible wirings of the ENGRO1 model, we newly identified and rationalized intense aerobic glycolysis and glutamine reductive carboxylation as the fittest emergent strategy that supports cancer cell growth under conditions reported to be generated by oncogenic activation: sustained glucose and glutamine uptake. This emergent phenotype is observed only when the available carbon exceeds the amount that could be fully oxidized by the available oxygen, a condition that is entirely physiological. We validated our predictions by analyzing the metabolic behavior of murine *K-ras* transformed fibroblasts, grown in high glucose together with either low or high glutamine availability.

## Results

### ENGRO1 metabolic network reconstruction

In the context of FBA, nutritional conditions are simulated either by imposing a specific *uptake flux* or by specifying the maximal rate allowed for nutrient consumption reactions. We will refer to the latter case as a constraint on nutrient availability, bearing in mind that it should be interpreted as a constraint on intracellular rather than on extracellular nutrient availability, the relationship between the two being non-linear and controlled by complex signaling networks [31].

Given that the actual consumption rate of each individual nutrient *in vivo* is undetermined, we wanted to systematically evaluate the typical response of a human metabolic network to different boundary conditions. For the sake of feasibility, we limited our analysis to glucose and glutamine—the most abundant nutrients in plasma [32] as well as the major cancer nutrients—disregarding the metabolism of other minor carbon sources.

**Fig 1:**
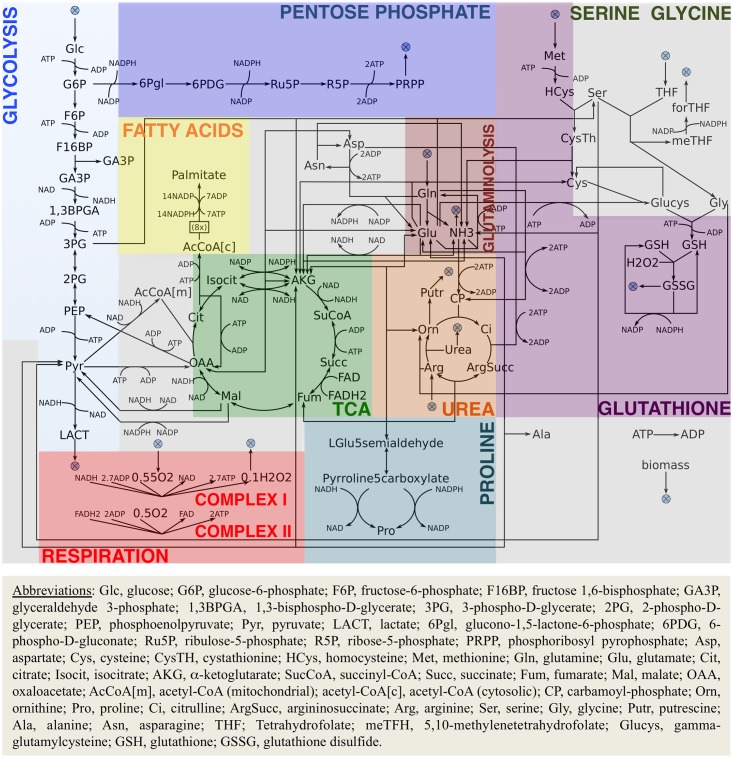
ENGRO1 model. Pathways and reactions included in ENGRO1. A crossed circle symbol indicates exchange reactions.

The metabolic network ENGRO1, designed to evaluate the contribution of glucose and glutamine to biomass formation, was therefore manually reconstructed. The network includes the catabolic pathways of glucose and glutamine and the anabolic reactions necessary for the main building blocks of biomass: amino acids and fatty acids (palmitate), together composing 80% of the dry cellular weight [23]. We focused on the pathways that are known to be utilized by cancer cells as reviewed in [20] and depicted in Fig 1. Reaction stoichiometry and directionality was taken from genome-wide databases Recon 2 [23] and the HMR [24]. When conflicts were detected between the two sources, we consulted the KEGG database [33, 34]. We manually handled feasibility problems of model simulations, based on literature. To streamline the analysis of ENGRO1 emergent properties, we lumped those reactions that necessarily operate together, a common practice in metabolic network modeling [35]. With one exception, all metabolites and reactions belong to a single, lumped intracellular compartment that includes cytosolic, mitochondrial and membrane reactions. Only Acetyl-CoA has two separate pools, one pool being devoted to fatty acid synthesis. The obtained model is structurally free from thermodynamically infeasible loops, a recognized problem in genome-wide models [36].

### Biochemical properties of flux pattern ensembles able to promote growth

Although cancer cells proliferate under conditions that restrain growth of normal cells, their growth rate does not necessarily coincide with the global optimum: indeed, it has been argued that a 5% improvement in growth rate would suffice to provide a selective advance to cancer cells [32]. We have little knowledge about the different ways in which a cancer cell may obtain a selective advance: however, we can deduce the probability distribution for all the allowed states, from a few boundary conditions.

Instead of focusing on the optimal response to a nutrient change (standard FBA), we examined the typical properties of the ensemble of theoretical wirings whose generic properties statistically match those of cancer cells, an approach similar to the one first applied to the study of gene regulatory networks [37]. To effectively sample the space of admissible wirings, we exploited the method proposed in [38] based on multi-weighted random objective functions. In biological terms, each wiring represents a virtual cell within a population of randomly altered gene expression profiles. By way of example, genetic or epigenetic activation of a gene encoding a metabolic enzyme would correspond to an attempt to increase the flux through that enzyme, while the mutational activation of a regulatory gene should do so for a set of such fluxes.

Because the precise boundary conditions of cancer cells *in vivo* and *in vitro* are largely variable and mostly unknown, with special regard to oxygen consumption rates, we simulated the response of 50,000 wirings to a modulation of either the glucose or glutamine uptake flux, while the maximal oxygen consumption rate (i.e., oxygen availability) was kept constant across all the experiments.

**Fig 2:**
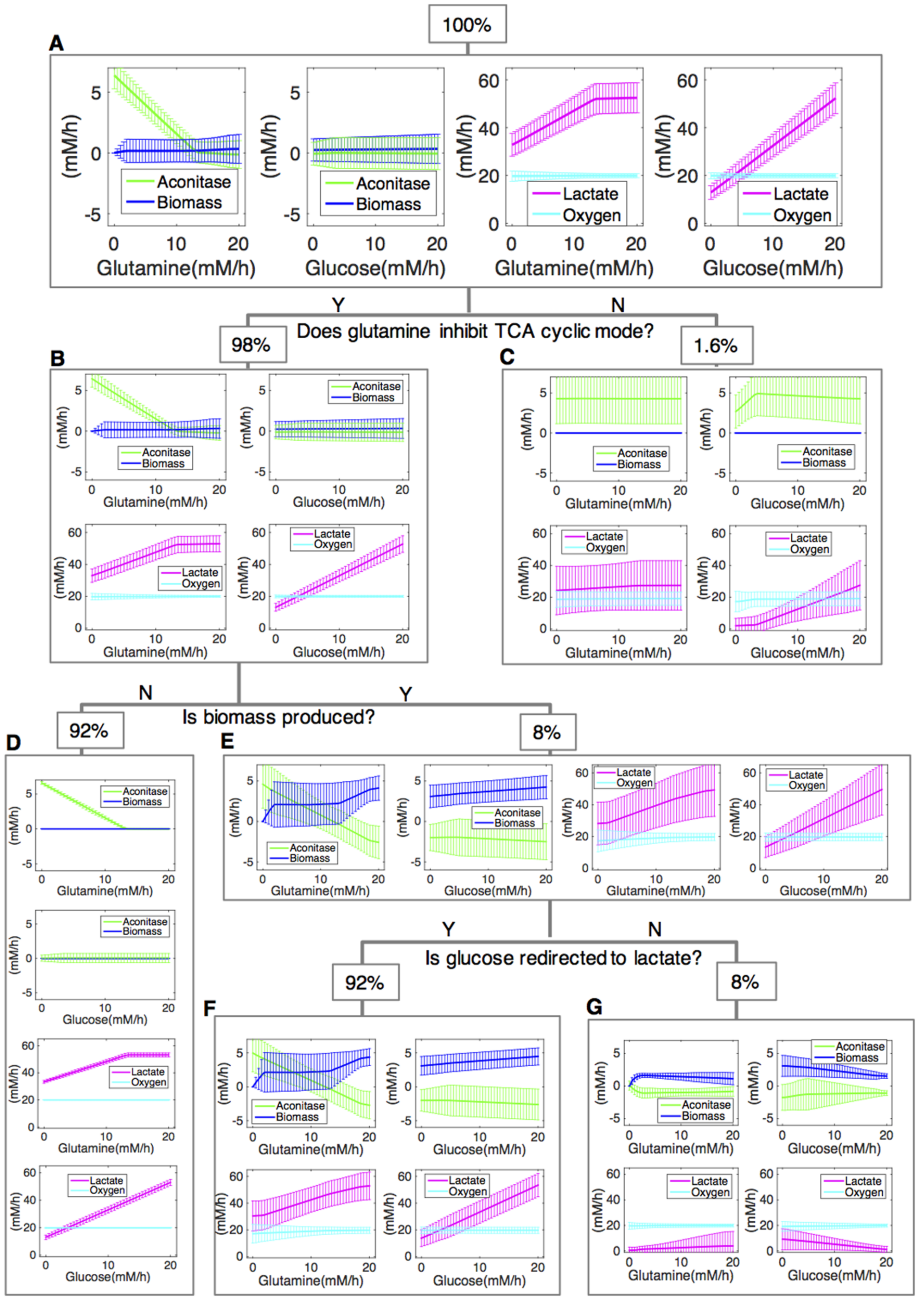
Ensembles of different metabolic responses to altered boundary conditions. Different uptake fluxes are imposed for glucose and glutamine within the range [0, 20]. While varying one parameter, the availability of the other is left at its baseline value (G:20 mM h^-1^; Q:20 mM h^-^1; O_2_:20 mM h^-1^). For each parameterization, the set of 50,000 multi-weighted objective functions is optimized. For the total sample (panel A) and for the different identified subsets (panels B-G), the average flux value of aconitase (green), biomass synthesis (blue), lactate secretion (magenta) and oxygen uptake (cyan) is reported as a function of glutamine (left or top insets) or glucose (right or bottom insets).

Although the 50,000 *in silico* cells displayed considerable variability in flux patterns, Fig 2A shows that certain patterns emerged: for instance, wirings simulated with higher carbon availability tended to produce more lactate. Strikingly, despite the constant level of oxygen consumption, we observed a decrease in the rate of the forward aconitase reaction (citrate to isocitrate), with increasing glutamine uptake rates. The aconitase forward flux is a proxy of the operational activity of the TCA cycle as a cycle.

Despite the high utilization of nutrients, ENGRO1 wirings on the average do not exhibit biomass production (the median and mode value of the biomass production fluxes are 0). Therefore, lacking a prerequisite for cell proliferation, these simulated cells would not be classified as tumorigenic (Fig 2A). The distinguishing properties of this non-growing, as well as of other ensembles, can help to uncover the design principles of metabolic growth.

We thus partitioned the total population into distinct sub-populations that respond differently to nutritional conditions. Based on the interest raised by aerobic production of lactate, a first filter was applied to investigate the mode of utilization of glutamine: the cyclic mode of TCA cycle was reduced by glutamine in 98% of cell types (Ensemble B in Fig 2B). Less than 2% of cell types show a nearly constant aconitase flux with a null growth rate (Fig 2C). We dissected the variability in biomass production rates of Ensemble B (Fig 2B), filtering out more than 90% cells that do not grow (Ensemble D—Fig 2D). When comparing proliferative against non-proliferative wirings, we realized that reversion of the aconitase flux (citrate to isocitrate) from positive (forward) to negative (backward), as the glutamine flux increases, is only detectable in the subset of growing cells (Ensemble E—Fig 2E), indicating that exploitation of reductive carboxylation of glutamine supports cell growth. A non-negligible fraction of the growing cells (8%, Fig 2G), did not show redirection of glucose to lactate: therefore, the increase in the glycolytic to oxidative ATP flux ratio (AFR) (27) that has been associated with the Warburg effect [39] is not a necessary condition for growth. In the large majority of cases (92%) reductive carboxylation of glutamine accompanies the redirection of glucose to lactate (Fig 2F), indicating a link between these two phenotypes.

Taken together, results in Fig 2 indicate that, despite identical boundary conditions, only a subset of wirings succeeds to grow, being characterized by the ability to utilize glutamine through a reverse aconitase flux and to produce lactate.

### Predicted ENGRO1 behavior in limiting-glutamine is experimentally verified in cancer cells

**Fig 3:**
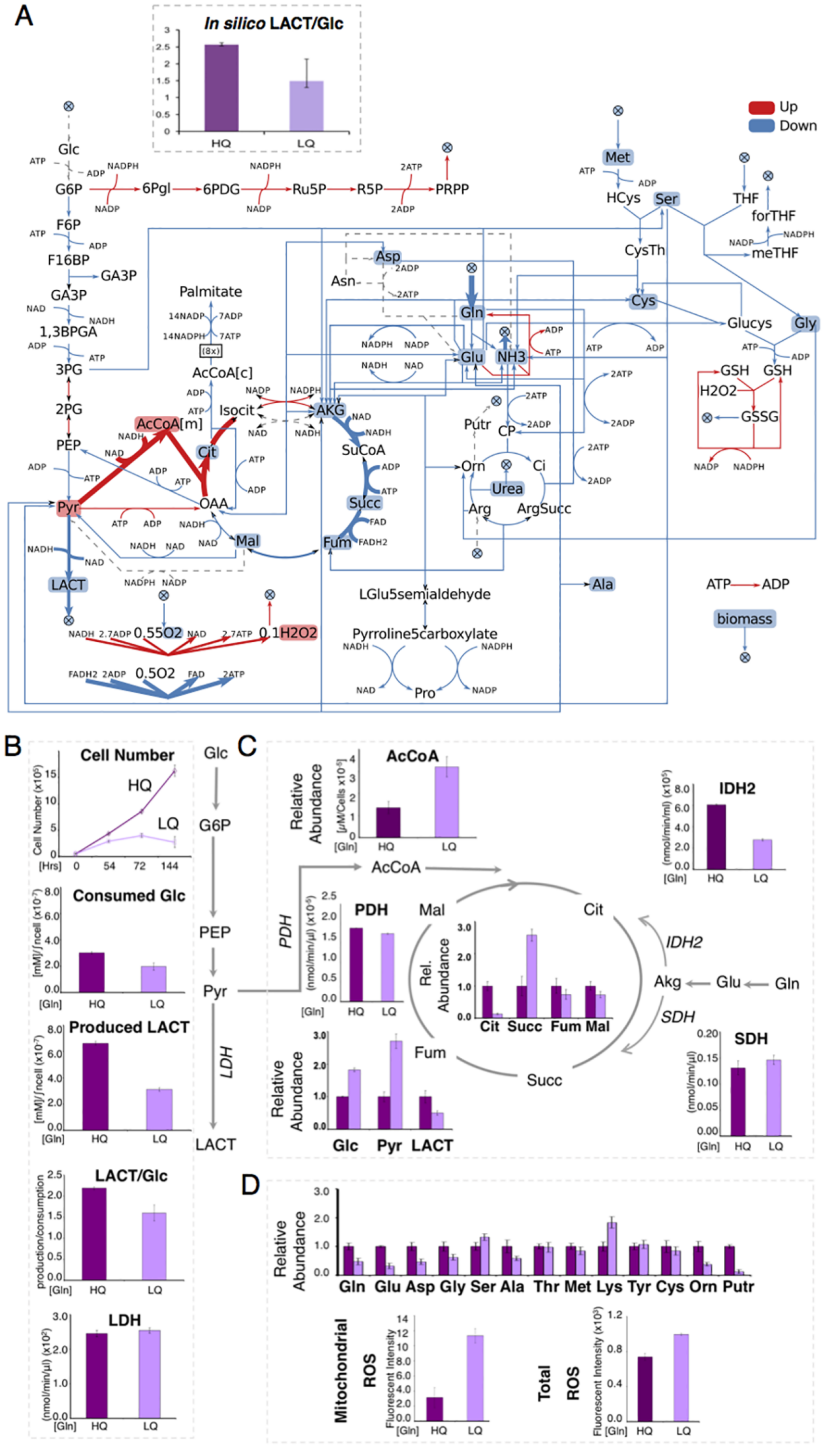
ENGRO1 and NIH-Ras mouse fibroblast sensitivity to glutamine limitation. A) Sensitivity of Ensemble E models to a reduction in glutamine availability from 20 to 2.5 mM h^-1^. Red arrows/boxes correspond to statistically significant increased fluxes/metabolites (p-value<0.05), blue arrows to decreased fluxes/metabolites; dashed-grey arrows to non-significant variations. Variations in fluxes always refer to the forward net flux. The arrow thickness is proportional to the Z-score. For the ratio LACT/Glc (production/consumption) average and standard deviations are reported. (B) Proliferation curve of NIH-Ras mouse fibroblasts grown in 4 mM Gln (HQ) and 0.5 mM Gln (LQ). The cells were collected and counted at the indicated time points. The glucose and lactate absolute quantifications in spent media, and the ratio between the two, were performed by GC-MS. Lactate dehydrogenase (LDH) enzyme activity was measured by enzymatic assay. (C) Intracellular relative metabolite abundances of AcCoA, LACT, Glc, Pyr, Mal, Succ, Fum measured by GC/MS. Pyruvate dehydrogenase (PDH), succinate dehydrogenase (SDH) and isocitrate dehydrogenase (IDH2) enzyme activity. To compare more easily the magnitude of change induced by the low glutamine condition, the concentration of each metabolite in the high glutamine condition is always taken as 1.0, regardless of it absolute value. (D) Relative metabolite abundance of non-essential amino acids as analyzed by GC/MS. Intracellular total ROS levels measured by using 5 mM DCFH2-DA staining. Mitochondrial ROS levels measured by MitoSOX Red mitochondrial superoxide indicator. To compare more easily the magnitude of change induced by the low glutamine condition, the concentration of each metabolite in the high glutamine condition is always taken as 1.0, regardless of it absolute value. All the experiments were performed on NIH-Ras grown in 4 mM Gln and 0.5 mM for 144 h. Error bars indicate the standard deviations (n = 5).

The proliferative wirings identified above are heterogeneous, but share some generic properties: we thus tested whether they could also be found in a cancer cell line grown in similar nutrient conditions. Fig 3 compares experimental results obtained by growing *K-ras*-transformed NIH-3T3 murine fibroblasts [40, 41], in media supplemented with either high or low glutamine (Gln), following the same experimental protocol reported in [42], against the alterations in fluxes that were computationally predicted for proliferative wirings (Ensemble E), under conditions of high or low glutamine uptake (Fig 3A).

To increase the sample size of Ensemble E, we constrained biomass to be produced and simulated a new sample of 50.000 random objective functions at high and low glutamine. Growth and metabolism of both the *in silico* cells (Fig 3A) and the cell line (Fig 3B and 3C) showed glutamine dependence.

We could quantitatively validate the decrease in the ratio of lactate produced to glucose consumed at low glutamine. The model also correctly predicted that the lactate/glucose ratio exceeds 2 at high glutamine (Fig 3A), indicating that some glutamine is converted to lactate.

Attempts to include information on metabolite levels into FBA computations, by allowing a set of metabolites to escape the steady state assumption, have been proposed [23, 30, 43, 44]. Along with biochemical interpretations, we thus repeated the 50.000 simulations in Fig 3A, adding a virtual efflux reaction for the set of experimentally measured metabolites. If the flux through the virtual efflux reaction was significantly increased in low glutamine, we indicated the corresponding metabolite as increased (Fig 3A); if the flux was significantly decreased, we marked the metabolite as decreased.

We correctly predicted an increase in pyruvate, acetyl-Coa and hydrogen peroxide (H_2_O_2_): the experimental concentration of these metabolites is indeed increased in low glutamine (Fig 3C and 3D).

Besides, the TCA intermediates Citrate, α-ketoglutarate, Malate, Fumarate are correctly predicted to be down-regulated in low glutamine. Our prediction of a decrease in succinate level did not match the experimental result (Fig 3C). The inaccurate prediction for this metabolite may be due to intrinsic limits of FBA, which is not able to capture regulatory effects [45, 46], such as the responsiveness of Complex II (succinate dehydrogenase) respiration flux to changes in the ATP/ADP ratio (see S2 Text for a more accurate but complex tentative prediction).

We correctly predicted the decreased level of most amino acids (glutamate, aspartate, glycine, alanine, methionine, cysteine), but we failed to predict the slight increase in the level of serine. This minor inconsistency may derive from the fact that in our simulation we did not supply external serine.

We did not predict ornithine and putrescine to be significantly changed because these metabolites display a large variability in our sample. Efflux of ornithine and putrescine are indeed energetically equivalent in ENGRO1 network (cofactors are not involved in the step from ornithine to putrescine). We did not predict glucose to be significantly changed, as experimentally detected.

Hence, the extended computational analysis was able to capture with a good degree of accuracy how the levels of many metabolites respond to glutamine shortage, giving support to the reliability of the ENGRO1 model and of our ensemble approach. A similar pattern of response has been obtained by a biochemical interpretation of the experimental findings (see S2 Text).

### Sustained glutamine utilization makes aerobic glycolysis optimal for cancer growth

In the previous sections we used a random sampling approach coupled with parameter scan of glutamine and glucose uptake rates to highlight that in the presence of glutamine there is a trend (for many but not all sampled objectives) to increase the amount of glucose that is converted to lactate. After having validated most model predictions using a murine cancer cell line, in this and the following sections we use standard FBA to understand, from a theoretical point of view, if the preference for glutamine is related to ATP and/or biomass optimization and how this preference is affected by the boundary conditions, i.e. the relative availability of oxygen, glucose and glutamine.

**Fig 4:**
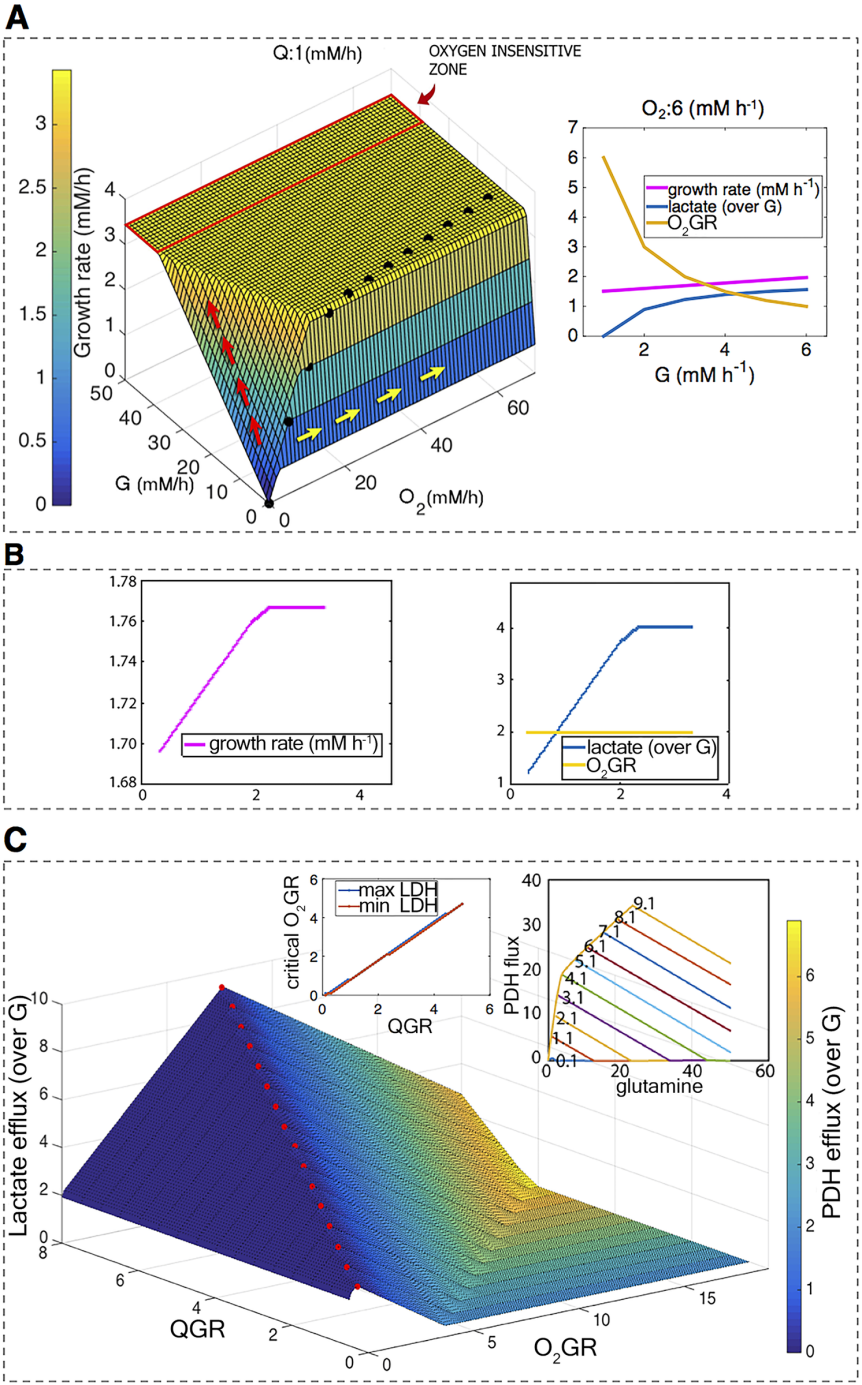
ENGRO1 biomass optimization for different boundary conditions. A) ENGRO1 maximal growth rate (*z-axis* and coloring) as a function of glucose (G) and oxygen availability when glutamine availability is constant (Q:1). The arrows represent example mutational paths in the fitness landscape. The insets represent 2D-slices of the 3D mesh when oxygen availability takes value 6. B) The growth rate (over G) and lactate secretion (over G) as a function of glutamine availability (indicated as the glutamine over glucose ratio QGR), when the O_2_GR takes value 3 (O2:6 (mM h-1), G:3 (mM h-1)). C) Lactate efflux (z-axis) and PDH flux (color scale) scaled on glucose uptake as a function of QGR and O_2_GR. Red points highlight the O_2_GR level for which maximum lactate secretion is observed. Left inset: level of oxygen to be considered critical as a function of QGR. Critical oxygen is computed both as the point in which lactate secretion is at the maximum (blue dots) and the point in which PDH reaches its minimum (red dots). Right inset: pyruvate dehydrogenase flux as a function of glutamine availability (QGR) for different oxygen availability values (the O_2_GR value is reported on top of the corresponding curve).

First, we analyzed the situation in which glutamine is supplied in a small quantity, just sufficient to provide the nitrogen for growth (Fig 4A). In this condition, the interplay between oxygen, glucose and growth behaves as expected: i) in a situation of fully oxidative metabolism–stoichiometrically given by an oxygen-over-glucose availability ratio (O_2_GR) of at least 6 (black dots in Fig 2A)—an increase in glucose uptake (red arrows in Fig 4A) provides a considerable improvement of growth. On the contrary, an increase in oxygen (yellow arrows in Fig 4A) has no effect on growth, since at any O_2_GR over 6 it can fully oxidize the available glucose; ii) all extra glucose supplied will decrease the O_2_GR and will thus be fermented to lactate (Fig 4A inset). iii) the higher the glucose consumption, the lower the sensitivity of the growth rate to a decrease in oxygen availability (red rectangle in Fig 4A). This last property is worthy of consideration: the established up-regulated glucose consumption of cancer cells might already suffice to make the growth of cancer cells less dependent on oxygen than the growth of normal cells.

The relationship between oxygen availability and glucose fermentation becomes more complex when glutamine enters the equation. If we set the O_2_GR, for instance, to 2, the provision of extra glutamine, despite providing an appreciable advantage in terms of growth (Fig 4B, left), comes at the cost of further glucose diverted to lactate (Fig 4B, right). Indeed, we show in the insets of Fig 4C that glutamine decreases the pyruvate dehydrogenase flux and increases the fraction of glucose fermented to lactate expected for a given O_2_GR, thereby making aerobic glycolysis optimal for growth. Fig 4C reports the lactate efflux and the pyruvate dehydrogenase (PDH) flux of the optimal growing cell as a function of glutamine and oxygen uptake. A peak in lactate secretion is detected in correspondence with non-negligible values of oxygen and with a null PDH flux, indicating that no pyruvate is converted to acetyl-CoA and thus all oxygen is utilized to partially oxidize glutamine and none to fully oxidize glutamine or glucose to CO_2_. For any value of glutamine availability, a corresponding O_2_GR level for which all glucose is redirected to lactate could be identified (inset in Fig 4C). We refer to this O_2_GR level as critical O_2_GR. A similar metabolic rewiring is remarkably observed at critical O_2_GR, regardless of the corresponding QGR (glutamine over glucose availability) value (S1 Fig). Results are also robust to variations in the amino acid composition of proteins (S1 Fig).

Notably, although we simulated the modulation of oxygen consumption by altering the boundary on oxygen intracellular availability, comparable effects were observed when constraining the capacity of Complex I to carry flux, and thus to accept NADH electrons, to mimic mitochondrial dysfunction (S2 Fig).

### CMR is characterized by a branched TCA cycle and coincides with the fittest growth-promoting wiring

Although cancer cells divert most—but not necessary all—glucose to lactate, to better understand the principles governing the utilization of glutamine carbon in cancer cells, we focused on the extreme condition identified above in which all glucose is preferentially redirected to lactate (critical oxygen condition identified above). Without loss of generality, we arbitrarily picked a critical oxygen boundary conditions. Notice that similar conclusions would be derived for different values of the ratio between glucose and glutamine consumption, by accordingly adjusting the utilization of oxygen (S1 Fig).

**Fig 5:**
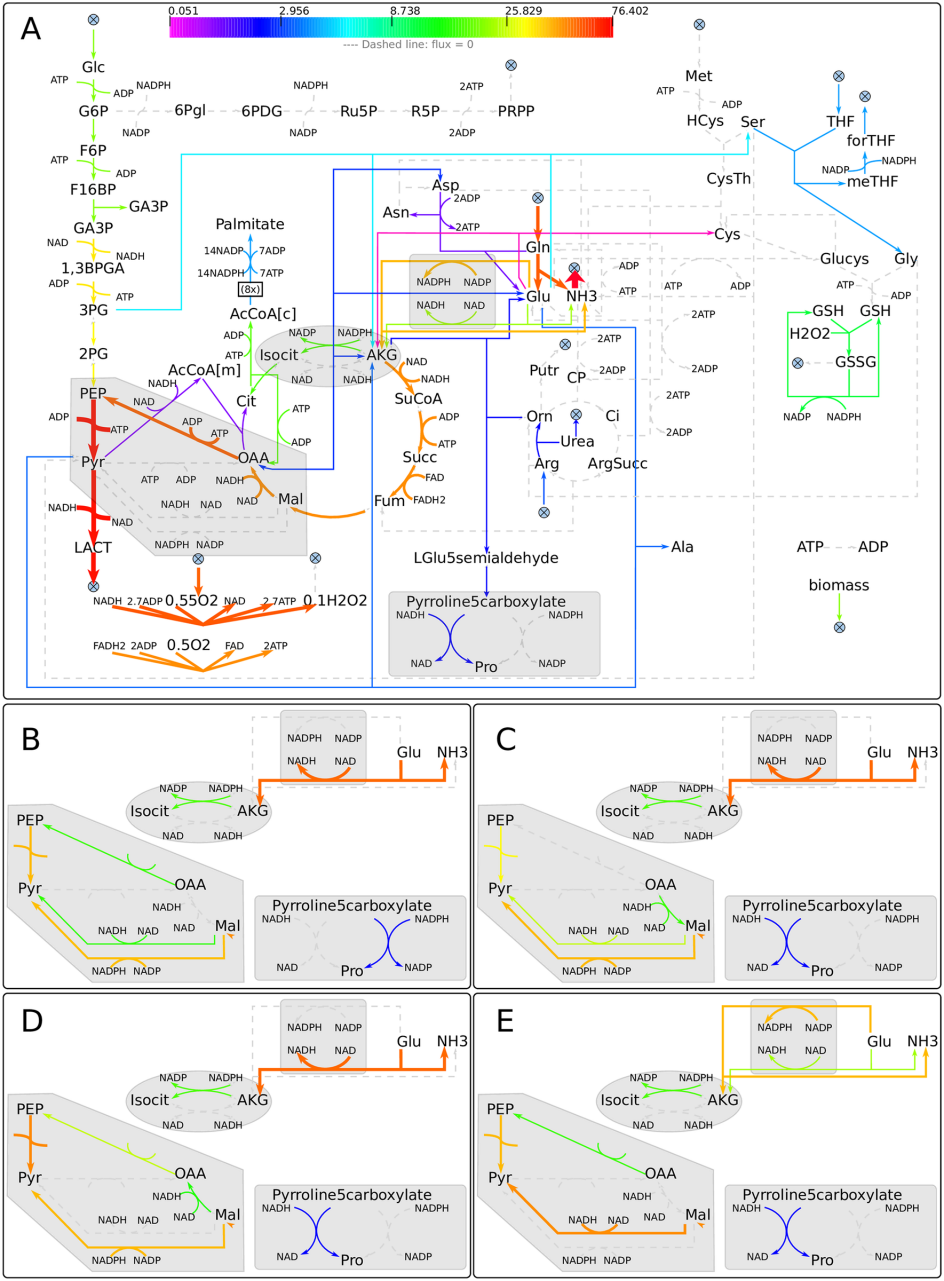
Flux patterns at critical O_2_GR. A) ENGRO1 flux distribution that maximizes growth at critical O_2_GR (O_2_: 38 mM h^-1^; G: 10 mM h^-1^; Q: 40 mM h^-1^) as per S1 Table. The color and thickness of arrows scale with the flux intensity. Dashed grey lines are associated with null fluxes. The direction of the flux is indicated by the point of the arrow that is colored. Shaded shapes encompass reactions that show variability in optimal solutions. B-E) Examples of alternative flux patterns in optimal solutions.

Fig 5A reports a representative optimal flux distribution at critical O_2_GR. It can be observed that the TCA “cycle” works in a branched non-cyclic mode, in which the α-ketoglutarate (AKG) originating from glutamine (virtually all glutamine is converted to glutamate and then to AKG) takes a reverse path to isocitrate and then fatty acid biosynthesis (for about 20%) and a clockwise path to malate (for about 80%) which is eventually converted to pyruvate and then lactate. Excess TCA intermediates not contributing to biomass (including part of the OAA derived from the synthesis of citrate that fuels fatty acid biosynthesis) are preferentially disposed *via* conversion to lactate. The pentose-phosphate pathway is not active, reflecting the fact that our model does not account for synthesis of nucleic acids, a small fraction of cellular biomass. The electron transport chain is nearly equally distributed between oxidation of FADH and NADH, which also generates hydrogen peroxide (H_2_O_2_). H_2_O_2_ is totally removed by the coordinated action of glutathione peroxidase and glutathione reductase, with NADPH serving as an electron donor, despite the possibility to remove H_2_O_2_ without energy costs *via* a demand reaction. Besides lactate, another major toxic product is excreted: the ammonia that is not used for protein synthesis is removed as such, without using the ATP-costly urea cycle.

The overall flux pattern that maximizes growth, hereinafter referred to as branched TCA cycle even though TCA is not working as a cycle, is in line with literature reporting experimental findings. Conversion of most glucose to lactate in spite of oxygen availability has been observed in many cancer cell lines or tumors [6, 7], as well as decoupling of glucose and glutamine pathways, with glutamine largely sustaining biomass production [19, 47, 48], and excess nitrogen elimination through sustained ammonia efflux [49–51]. Reductive carboxylation of glutamine has been recognized to drive fatty acid synthesis [15, 17, 19, 52]. Glutamine was identified as the major respiratory fuel [13, 53, 54], while lactate derivation from both glucose and glutamine has been reported for MYC-dependent cancer cells [49]. As far as we know, there is no previous direct attempt to evaluate the lactate over glucose ratio at low and high glutamine. However, results obtained using a more severe nutritional perturbation (i.e., glutamine starvation on six tumor cell lines able to grow in the absence of glutamine) are qualitatively consistent with the predictions of our model. In the only cell lines in which the ratio between lactate produced/glucose consumed is affected by more than 10% in the absence of glutamine, the observed change in the lactate produced/glucose consumed ratio is decreased in glutamine-depleted cells [55]. Finally, reduction of oxidation of glucose carbon entering the TCA cycle by glutamine has been reported [56]. In conclusion, literature data reinforce the notion that the behavior observed in our model cell line and the predictions of our computational model do not refer to a specific case, but describe a metabolic rewiring that may occur in a variety of tumor cell lines, putting forward the idea that this competition between glucose and glutamine may play a determinant role in cancer cell growth.

### CMR may be controlled by various redox pathways

We examined if the flux pattern in Fig 5A was the only possible one for the fittest cell.

When enumerating optimal solutions, we found that 44 alternative metabolic paths, sharing the same boundary conditions, produce the same maximal growth rate. Remarkably, all of them follow the branched TCA cycle mode described above.

It has been already reported that variability in the optimal solution space is captured by a small number of sub-networks constituted by reactions with variable flux [57]. Accordingly, only the four sub-networks shaded in Fig 5A show alternative flux patterns (examples in Fig 5B–5E). For instance, the sub-network within the shaded polygon (in Fig 5A), diseregarding pyruvate carboxylase, includes: phospho-enol-pyruvate carboxykinase; pyruvate kinase, NADP and NAD dependent malic enzyme and malate dehydrogenase. Because the TCA cycle is working in a non-cyclic mode, this set of reactions allows to maintain the steady state by removing glutamine-derived TCA cycle intermediates: oxaloacetate (OAA) which is derived from the citrate, produced by glutamine-dependent reductive carboxylation, the co-produced AcCoA being utilized for fatty acids biosynthesis and malate deriving from fumarase, which, by means of the reactions in this subnetwork, can be converted into pyruvate and then secreted as lactate. The more relevant options to remove OAA and malate are reported in Fig 5B–5E. Note that malate dehydrogenase and PEP carboxykinase fluxes are completely positively correlated (S3 Fig): when PEP carboxykinase is off, malate dehydrogenase must work in the backward mode (reduction of OAA to malate); on the contrary when malic enzyme is off and malate dehydrogenase works to oxidize malate to OAA, PEP carboxykinase must carry all the flux from TCA to pyruvate. Another group of reactions allowing for metabolic plasticity (shaded oval in Fig 5A) is composed by NAD dependent and NADP dependent isocitrate dehydrogenase (IDH), which both can convert AKG to Isocitrate. As both reactions are reversible, different modes are possible to obtain the same net amount of isocitrate. Note that NAD-IDH and NADP-IDH never work in the same direction in optimal solutions, as confirmed by the complete negative correlation between the two (S3 Fig).

It can be observed that the four sub-systems involve redox factors and may therefore influence one another (S3 Fig). For instance, when the isocitrate dehydrogenase sub-network is working in the NADPH to NAD+ trans-hydrogenation mode, the other sub-networks must provide the NADPH needed, as shown by the strong anti-correlation between NADP-dependent IDH and the sum of the fluxes of NADPH producing reactions: glutamate dehydrogenase and malic enzyme (S3 Fig). Along similar lines, when the flux of NAD-IDH is in the NAD generation mode (negative flux from isocitrate to citrate), needed NADH is partially produced by GDH and malic enzyme (as suggested by the partial negative correlation between NAD-IDH and NADH production by GDH and malic enzyme in S3 Fig). Notably, (i) the net NADPH produced by NADP dependent malic enzyme, glutamate dehydrogenase and IDH and the fixed amount produced by MTDH1 always equals that consumed by fatty acids biosynthesis, glutathione oxidase and proline biosynthesis and (ii) the net NAD produced by NAD dependent malic enzyme, glutamate dehydrogenase and IDH and by malate dehydrogenase must match the quantity of NADH required to feed Complex I respiratory chain, lactate dehydrogenase and proline synthesis.

A role for redox control in CMR has been anticipated in [20]. The presently observed variability in redox patterns is interesting since it may provide a novel partial explanation for the reported metabolic plasticity of cancer cells [58], which would be generated by genetic and epigenetic variations that lead to the emergence of distinct subpopulations that were selected on the basis of an enhanced growth rate. Redox reprograming deserves more in-depth investigations in the future with compartmentalized and more extended models.

### Glutamine to lactate yields more ATP per O_2_ as compared to glucose to CO_2_

We have shown that glutamine utilization makes aerobic glycolysis optimal for growth. Nevertheless, the results of our ensemble approach (Fig 2) indicated that glutamine addition inhibits glucose oxidation in almost all simulated cells (either proliferative or not), regardless of the gene-expression dependent active wiring. How can a network structure constrain the flux pattern that strongly? A possible explanation is that most random metabolic functions have ATP production as co-objective to counterbalance ATP-demanding reactions.

**Fig 6:**
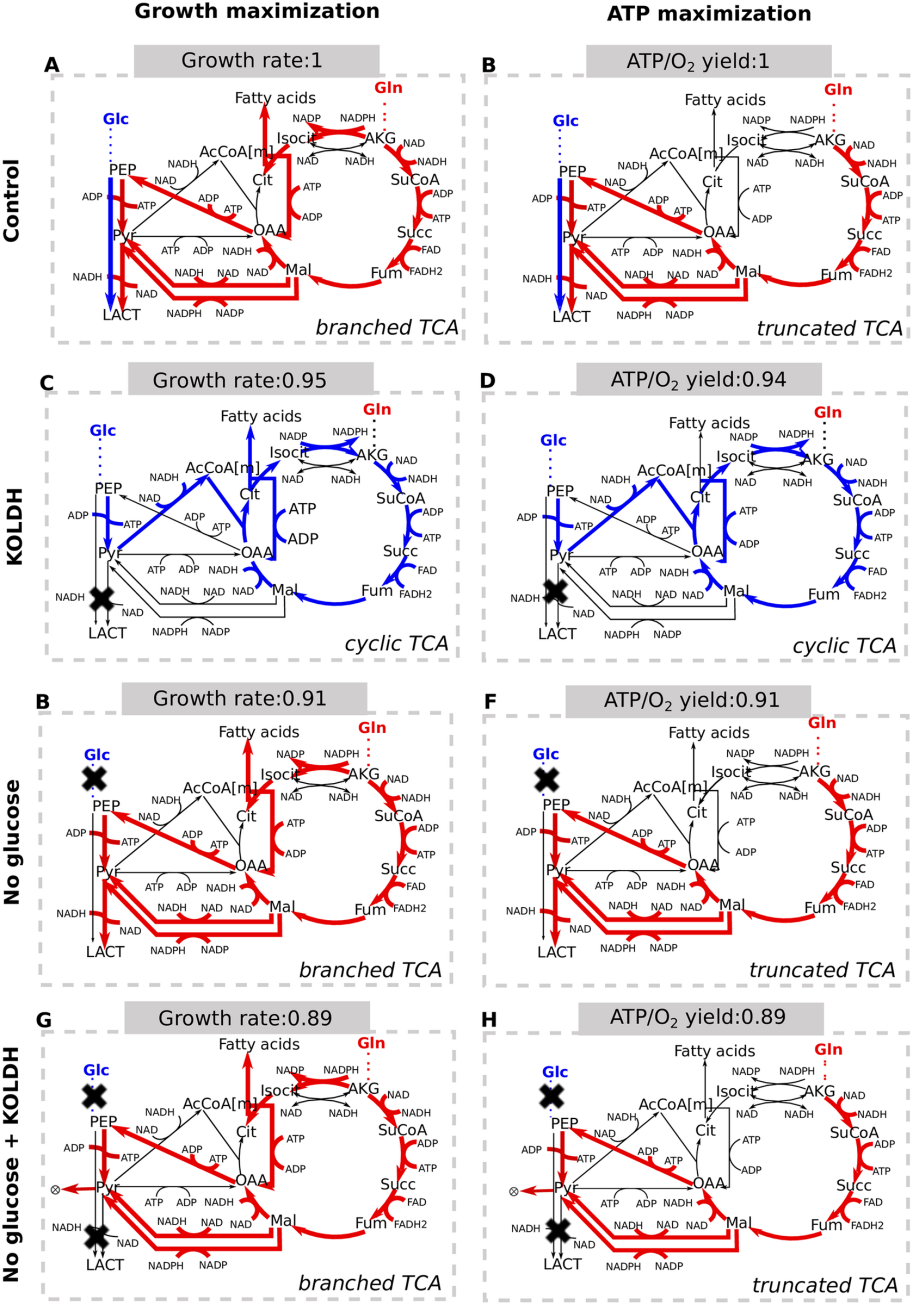
Qualitative representation of the TCA cycle flux mode associated with different optimization problems. Red arrows indicate carbon fluxes deriving from glutamine, while blue arrows indicate carbon fluxes deriving from glucose. For each optimization, the value of the objective function value is reported on top of the scheme and it is normalized over the corresponding objective value of the control model. Left panels refer to the solution of biomass maximization problems, whereas right panels refer to the solution of ATP maximization problems. A-B) Optimal solutions of control model (O_2_: 38 mM h^-1^; G: 10 mM h^-1^; Q: 40 mM h^-1^). C-D) Optimal solutions when lactate secretion is inhibited. E-F) Optimal solutions when no glucose is available. G-H) Optimal solutions when no glucose is available, lactate production is blocked and pyruvate is allowed to accumulate.

To investigate this hypothesis, we compared the previously obtained flux distribution at critical oxygen that maximizes growth (Figs 5A and 6A) against the one maximizing ATP production (namely the maintenance reaction flux). Consistently with the hypothesis, also the latter showed no oxidation of glucose (Fig 6B). As expected it does not fully utilize glutamine, conversely it makes use of only 63% of available glutamine (S2 Table), it shows null growth and no reductive carboxylation of glutamine. It follows that the TCA cycle is not branched but works in a truncated mode.

We wanted to verify that a cyclic TCA mode was still possible for ENGRO1 at critical oxygen, so we forced it by inhibiting lactate secretion (Fig 6C and 6D). In this situation glucose was preferred as anaplerotic precursor, resulting in a modest drop in both biomass formation and ATP production.

Noteworthy, the preference for a branched TCA cycle is maintained when glutamine is the unique carbon source (Fig 6E and 6F), even when lactate secretion is prevented (Fig 5G and 5H), provided that pyruvate accumulation is allowed. These results suggest that a branched TCA may be compatible also with glutamine carbon tracer experiments in which lactate is found unlabeled.

Taken together these observations indicate that glutamine is the preferred precursor for biomass production because it optimizes the ATP/O_2_ yield. The glutamine to lactate flux mode improves the ATP production over oxygen consumption ratio by 5% as compared to the glucose to CO_2_ mode. Glutamine to lactate coupled with glutamine driven fatty acids synthesis improves the biomass production rate by 5% with respect to glucose to CO_2_ and glucose driven fatty acids synthesis. The correlation between lactate secretion and the ATP cost to make biomass further supports this contention (S4 Fig).

The strength of this preference for glutamine seems to depend on the ATP/oxygen ratio (P/O) ratio for FADH and NADH, and thus on the fraction of the electrons flowing through Complex I that ends up in superoxide anion (S4 Fig), as was also confirmed by analytical stoichiometric computations (S3 Table).

## Discussion

In this work, we exploited a novel constraint-based approach, based on the systematic evaluation of the possible wirings of the metabolic network required for the formation of biomass from glucose and glutamine, under different nutritional conditions. The first discovery presented in this paper is the identification of boundary conditions that taken together promote the emergence of cancer metabolic rewiring.

In the presence of an increased uptake of glucose and glutamine, the availability of intracellular oxygen is not sufficient to fully oxidize all carbon provided by both glucose and glutamine. This oxidative bottleneck is not given by the actual oxygen concentration per se, but rather by the ratio between the ability to consume oxygen and the available nutrients, i.e., glucose and glutamine. A condition in which there are less than 6 usable O_2_ molecules per glucose molecule may be typical for cancer cells. Detailed calculations—based on oxygen consumption rates reported in [59] and glucose consumption rates reported in [60] and in Fig 3—are extended in Materials and Methods. They indicate that even under the atmospheric conditions of our cell cultures, the oxygen consumption is not 6 times as much as that of glucose, therefore fitting within the boundary conditions described above. Also *in vivo* the oxygen to glucose ratio in the blood supply is at least 3 times less than the 6 required for complete respiration (see Materials and methods). The elevated glutamine consumption rates of cancer cells make this oxidative bottleneck even more effective. The oxygen concentration in peripheral tissues might be even much lower as conditions change dynamically between the periphery and the center of a tumor [61], as well as during its natural history. For instance, most solid tumors face a period of actual poor oxygen availability before neo-angiogenesis begins. During this period cells better able to cope with low oxygen conditions—or carrying mutations leading to a mitochondrial dysfunction limiting the ability of Complex I to accept electrons from NADH [62, 63] that would mimic actual oxygen limitation–are likely to be selected for. These cells would internalize more glucose than what can be fully oxidized. Indeed, various oncogenes have been reported to cause stimulated uptake of nutrients. In general, the rewiring here described should become advantageous for highly proliferating cells, such as cancer cells. Its associated possible drawbacks, such as the excretion of potentially toxic lactate and ammonia, would make it less favorable for normal, slowly proliferating or resting cells.

We have quantitatively shown that high glucose uptake rates have the side benefit of making growth rates less sensitive to oxygen variations. As most cancer cells result from mutation and selection, tumors might therefore have fixed, through mutations, advantageous phenotypes into their genotype. In such a situation with high mutation rates, the population will tend to lose (through mutations and thereby permanently) all activities it does not depend upon.

Following the agreement with a wealth of literature, as well as experimental validation, of main ENGRO1 model predictions, we analyzed the global *in silico* flux distribution that sustains CMR. We were able to integrate and rationalize the role of the various pathways observed in independent studies on cancer metabolism, as follows:

- Glutamine utilization follows a branched TCA cycle, which proceeds clockwise from glutamine/AKG to oxaloacetate and malate, or counter-clockwise from AKG to citrate, then to acetyl-CoA going to fatty acids plus oxaloacetate going to malate.
- Glucose is almost fully fermented to lactate even in the presence of oxygen.
- Glutamine is not completely respired, but it is rather converted to lactate or pyruvate.

By exploiting FBA, we investigated the relationship between different flux modes and their associated growth capability. We discovered that CMR is characterized by the following novel design principles:

- A large number of different metabolic routes may generate a CMR, by differential use of redox potential, while fatty acid synthesis gains a significant role as an acceptor of electrons, mostly coming from the assimilation of glutamine.
- Glutamine utilization promotes aerobic glycolysis, making the latter advantageous for growth, because it makes it energetically more favorable. This effect is enhanced by Reactive Oxygen Species (ROS) production by Complex I, with a stricter preference for the use of glutamine as an anaplerotic precursor when ROS production increases.

Aerobic production of lactate from glucose does not utilize all chemical potential of its substrate, thus it is generally regarded as unfit. Our results indicate that this is not the case. The phenomenon we observed *in silico* at limiting oxygen is not simply the fermentation of spare glucose, but a competition for oxygen between glutamine and glucose. The limiting amount of oxygen is preferentially used for the oxidation of the NADH and FADH2 generated when glutamine is converted to building blocks and lactate. As all available glucose is converted to lactate, we put forward a novel interpretation to what is classically understood as the ‘Warburg effect’.

As boundary conditions change, so do the flux distributions of virtual cells that allow–possibly sub-optimal—biomass accumulation under a variety of conditions, suggesting how cancer cells might be able to follow different metabolic routes *in vivo*, if the tumor environment imposes different constraints. In particular, we showed that fermentation of glutamine to lactate (that we call WarburgQ effect) is able to sustain growth even when glutamine is the exclusive simulated carbon source, suggesting how WarburgQ cells would be able to evade therapies that target only glycolysis.

In conclusion, our paper offers for the first time a “logic” to the central role of the metabolic hallmarks in cancer biology, logic that was envisioned in [4]. As the generalizability of predictions of metabolomics through FBA methods needs further research, it should be useful to engage in stable isotope-labeling studies and detailed mathematical analyses of the results to confirm our findings further. In the next-future, the general traits of cancer metabolic rewiring identified with our approach will be better detailed by means of more fine-grained models, taking into account compartmentalization and the role of metabolic shuttles, hence opening the way to more efficient approaches to drug discovery and to precision medicine not only for cancer, but also for other diseases in which a remodeling of metabolism has a relevant role, such as metabolic syndrome, ageing and neurodegeration.

## Methods

### ENGRO 1 model reconstruction

Once the pathways to be included were selected, we checked that each reaction in the model was able to carry flux. We included a minimal set of exchange reactions. We avoided compartmentalization of metabolites, paying attention that this simplification would not affect the energetic requirements for biomass. We associated a probability *p* to produce ATP and a probability *pROS = (1 − p)* to produce ATP to NADH oxidation in the respiratory chain. Biomass formation is expressed in terms of ATP, non-essential amino acids and palmitate requirements. Thermodynamically infeasible loops were first identified with the algorithm presented in [36] and then structurally removed by adjusting the metabolic network accordingly. All modeling choices are better detailed in S1 Text.

The resulting ENGRO1 model includes 84 reactions and 67 metabolites belonging to the following pathways: glycolysis and lactic acid fermentation (10 reactions); TCA cycle (9 reactions); oxidative phosphorylation and mitochondrial ROS generation (2 reactions); glutamine reductive carboxylation (4 reactions); glutathione biosynthesis and oxidation (10 reactions); urea cycle (5 reactions); fatty acid synthesis (2 reactions); pentose phosphate pathway (5 reactions) and biosynthesis of the main non-essential amino acids (13 reactions). The complete network definition together with the biomass composition is given in the S1 File (Excel file compatible with: RAVEN Toolbox [64]). The model is also available in RAVEN and FAME [65] compliant SBML format (S2 File). A vector image of the model is also provided (S3 File), which can be easily handled by FAME.

### Flux Balance Analysis (FBA)

FBA requires a stoichiometric matrix *S* and a set of constraints that impose the upper and lower bound of fluxes. The steady state constraint is defined by the equation *dx*/*dt* = *S* · *v* = *0*, where *dx/dt* are time derivatives of metabolite concentrations represented by the product of the *m*×*n* matrix *S* times the vector of fluxes *v* = (*v*_1_, *v*_2_, …, *v*_*n*_), where *v*_*i*_ is the flux of reaction *i*, *n* is the number of reactions, and *m* is the number of metabolites. The ensemble of functional states that the system can reach given a boundary condition *I* determines the feasible solutions space *Φ = Σ∩I*. By exploiting linear programming, FBA allows for optimization of the flux through a weighted sum of fluxes. In particular, we used the COBRA Toolbox [66] and the GLPK solver.

Because FBA only returns a single solution, we exploited Flux Variability Analysis [67] to assess the flux variation range consistent with the maintenance of the maximal growth obtained with FBA.

### Enumeration of optimal solutions

To analyze the complete set of flux distributions that maximize growth, we identified all the extreme points that lie at the intersection of the convex polytope given by the linear constraints and the hyperplane of the optimal objective function value, which correspond to alternate optima, as in [68]. The problem is formulated as a recursive Mixed Integer Linear Programming (MILP) problem that has a set of constraints for changing the basis and identifying a new extreme point corresponding to one of the alternate optima [68]. The method returns all the alternative paths in the metabolic networks that are consistent with optimal growth. Metabolic distributions that follow the same path, also in terms of reaction directionality, but with different flux values will not be taken into account.

### Sampling in the region of feasible solutions

As a complement to classic FBA, Monte-Carlo approaches are emerging with the aim of exploring the entire region of feasible flux distributions [69]. The dominant algorithm of choice to uniformly sampling inside the region of allowed solutions is the so-called "Hit-and-Run" [69]. In this work, we exploited instead a recently proposed alternative approach [38, 70]: the simplex method with a random set of objective functions to be maximized. The maximization of each of these objective functions gives a corner in the space of solutions. Besides what already reported in [70] another major advantage of sampling via random objective functions is the possibility to sample metabolic responses to perturbations [38].

### Multi-weighted random objective functions

In [70], random objective functions were generated by selecting random pairs of reactions. To maximize the variability of sampled solutions, we instead allow any number of reactions to take part in the OF as in [38, 71]. To further increase the variance of the corresponding set of flux distributions, when randomly assigning a weight to each flux in the computation of the different objective values, we introduce a bias towards 0. This prevents almost all fluxes to partake to any objective function, as this will result in more similar outcomes. We pick a different bias value for each sampled objective function.

The fraction *τ* of considered reactions is randomly drawn with uniform probability in (0, 1]. Any selected reaction is then assigned a random weight *c*_*i*_ that is uniformly tossed from the interval (0,1]. An instance *j* of the objective functions *z*_*j*_ is defined as $z_{j}=\sum_{i=1}^{n}c_{i}v_{i}$, where *c*_*i*_ takes value 0 with probability *τ* and a random value with uniform probability in [0,1] with probability *1-τ*.

Every *z*_*j*_ is assigned an optimal solution ${\hat{s}}_{j}$ with standard FBA.

### Assessment of deregulated flux after a nutrient perturbation

To determine the fluxes that were most sensitive to glutamine deprivation, we sampled the solution space of the model at control and at low glutamine conditions, exploiting the optimization of a random set of multi-weighted objective functions as in [38, 70]. For each flux, we then determined a Z-score that quantifies its significance of change and that can be positive or negative. *Z* is computed as $Z=\left(\bar{X_{1}}-\bar{X_{2}}\right)/\sqrt{\frac{\sigma_{1}^{2}}{n}+\frac{\sigma_{2}^{2}}{n}}$, where $\bar{X_{1}}$ is the average of the flux in condition 1 (high glutamine), $\bar{X_{2}}$ is the average of the flux in condition 2 (low glutamine), $\sigma_{1}^{2}$ is the variance for condition 1, $\sigma_{2}^{2}$ is the variance for condition 2, and *n* is the sample size.

### Determination of sample size

We chose *n* = 50,000 as a reasonable sample size, as greater samples would not result in a significant improvement in the estimation of the Z-scores nor of the standard error of the mean (S5 Fig), which takes value 0.1 at most.

### Ensemble analysis of different metabolic responses

To identify ensembles of metabolic responses abiding by specific constraints, we sampled many metabolic responses (corresponding to different objective functions) and then filtered them according to Boolean (true-false) expressions which must return true for each ensemble member. We define a metabolic response as the modification in fluxes resulting from a specific nutritional perturbation (e.g., glucose or glutamine consumption rate variation). The perturbation is simulated by varying the flux boundaries of the corresponding reaction. As an example, a metabolic response pertains to Ensemble E if and only if the biomass production flux is greater than 0 for at least one of the simulated glutamine and/or glucose uptake rates. For a more formal description of the method, the reader is referred to [38].

### Cell culture and proliferation

Mouse embryonic fibroblast *K-Ras*-transformed NIH3T3-derived cells [40–42, 72] were routinely grown in Dulbecco’s modified Eagle’s medium containing 4 mM L-glutamine, 100 U/μl penicillin and 100 mg/ml streptomycin supplemented with 10% newborn calf serum (complete growth medium) at 37°C in a humidified atmosphere of 5% CO_2_. Cells were plated at a density of 3000 cells/cm^2^ in 6-well plates in normal medium. Culture medium was replaced after 18 hours with a normal medium containing 4 mM Gln or a low Gln medium (0.5 mM glutamine). The cells were collected and counted after 54, 72 and 144 hours. Media and serum were purchased from Life Technologies Invitrogen (Carlsbad, CA, USA).

### Enzymatic assays

Kit for assay of acetyl-CoA and enzyme activities were purchased from BioVision (Milpitas, CA, USA) and used according to the manufacturer’s protocols. Raw data are provided in S4 File.

### Reactive Oxygen Species level measurements

The total ROS levels were measured using the DCFDA Cellular Ros Detection Assay Kit from Abcam (Cambridge, UK) according to manufacter’s instructions. Mitochondrial ROS levels were measured using 5 μM MitoSOX Red mitochondrial superoxide indicator (Molecular Probes) in HBSS/Ca/Mg for 10 min at 37°C. Fluorescence was measured at excitation/emission wavelengths of 510/580 nm, respectively, using a Cary Eclipse Fluorescence Spectrophotometer. Raw data are provided in S4 File.

### Intracellular metabolic profiling

After 144 hours of growth, the metabolites were extracted and analyzed by gas chromatography-mass spectrometry (GC-MS) as previously described [19] and the spent media were collected and filtered. 120-μ*l* of ice-cold acetonitrile was added to 40 μl of media and vortexed at 4°C for 2 minutes, and the mixture was incubated on ice for 5 minutes and centrifuged at maximum speed for 10 minutes. A 100-μl volume of the aqueous phase was collected in a new tube and evaporated under nitrogen flow at 37°C. Derivatization was performed as described [72]. A GC/MS analysis was performed as previously described. A 1-μ*l* volume of sample was injected in a 1:10 split mode at 250°C. The GC was performed as previously described [72]. The GC/MS data processing and metabolite quantification were performed using Agilent Muss Hunter software.

The following metabolites were detected: glutamine, glutamate, aspartate, glycine, serine, alanine, trehalose, methionine, lysine, tyrosine, cysteine, ornithine, putrescine, glucose, pyruvate, lactate, citrate, succinate, fumarate, malate, acetyl-CoA. Raw data are provided in S4 File.

### Calculation of the glucose over oxygen availability ratio of cancer cells *in vivo* and *in vitro*

According to the oxygen uptake rates catalogued for a variety of cells (including normal and cancer cells) in [59], the cellular oxygen consumption rate spans from 0.01*10^−13^ to 3.5*10^−13^ mMol per cell per second. We measured a glucose consumption rate of 5.8*10^−13^ mMol/sec per cell, while Jain et al. [60] reported values up to 2.10^−13^ mMol/sec per cell which would require 12.10^−13^ for complete respiration. Oxygen consumption is not 6 times as much as glucose, and thus–according to our terminology–an oxidative bottleneck is apparent for cancer cells growing *in vitro*.

The condition of limiting oxygen should also prevail in vivo. Let us approximately evaluate the molar ratio between oxygen and glucose in human blood:

- The concentration of oxygen in the blood (oxygen, dissolved plus hemoglobin bound, in 100 milliliter of blood) corresponds to a volume *V* of 20 ml of oxygen gas at a pressure *P* of 760 mmHg [73] and at a temperature of 310 Kelvin (*T* = 310K°)
- By using the ideal gas law P*V = n*R*T, where *R* is the universal gas constant 62 L*mmHg K^-1^*mol^-1^, we can obtain the moles *n* of oxygen per liter of blood.
- *n* = P*V/(R*T) = (760 * 0.2)/(62*310) ~ 8 mMol/liter.

Another procedure to compute *n* is to start form the hemoglobin concentration in blood, which we found in literature [73] to be 15g/100mL.

- Because the molar weight of hemoglobin is 64, the concentration of hemoglobin in blood is 2.3*10^−03^ mol/litre.
- If we assume a maximal saturation for oxygen, we have 4 oxygen molecules bound to each hemoglobin.
- Therefore, n = 2.3*10^−04^ *4 ~ 9 mMol/liter.

The two alternative procedures return a similar order of magnitude for the maximum concentration of oxygen in blood (in the range 8 to 9 mMol/liter). The level of glucose in blood is around 5 mol/liter [61]. Therefore, we can consider the oxygen-to-glucose ratio in blood to be closer to 1:1 than to 6:1.

Both glucose and oxygen are ultimately delivered to the tissue (extracellular environment) not by diffusion but by convection by the blood. Therefore, at maximum consumption of glucose from the blood, at most 2 molecules of oxygen would be available per glucose molecule. If one hypothetical cell were to consume avidly all the glucose that is supplied by the blood in a given unit of time, that cell would not have enough oxygen to completely oxidize such glucose in that unit of time.

## Supporting information

S1 Fig ENGRO1 biomass optimization for different boundary conditions (supplement to Fig 4). All data derive from Flux Variability Analysis in experiments that maximize biomass production. All reported fluxes do not show variability in optimal solutions. All glucose available is always fully consumed in these simulations. A) Growth rate (z-axis) and oxygen uptake (color scale) scaled on glucose uptake as a function of QGR and O_2_GR. The level of O_2_GR for which a plateau is reached (optimal O_2_GR) increases with the QGR (purple dots). For high O_2_GR a plateau is not reached within the considered range. S2 Fig show 2D slices of the 3D-mesh. B) Ratio between the flux trough Complex I and Complex II driven respiratory chain (z-axis and color scale) as a function of QGR and O_2_GR. Red dots highlight the value of the ratio when O_2_GR is critical. Full glutamine utilization takes place at Critical O_2_GR (panel C), when the PDH flux is 0 and the respiratory chain is fed exclusively by glutaminolysis leading to almost equal NADH (Complex I) and FADH (Complex II) oxidation, the ratio of NADH oxidation over FADH oxidation being around 1.2. C) Glutamine uptake (z-axis) and ammonia efflux (color scale)–scaled on glutamine availability and consumption respectively–as a function of QGR and O_2_GR. Red points highlight the minimum level of O_2_GR for which full glutamine consumption is observed. The increased glutamine uptake needs to be balanced by a comparable ammonia efflux that get closer to 2 when glutamine is plentiful and oxygen is low (coloring of the mesh). D) Aconitase flux (z-axis and color scale) as a function of QGR and O_2_GR. S2 Fig show 2D slices of the 3D-mesh. Red dots highlight the flux when O_2_GR is critical. At Critical O_2_GR, pyruvate dehydrogenase is not working, TCA cycle cannot work in a cyclic mode and production of citrate by the citrate synthase is therefore not possible. The citrate required to feed biosynthesis of fatty acids (required for biomass production) must derive from reverse carboxylation of glutamine derived AKG, as indicated by a negative flux of aconitase. E-H) The optimal flux value of serine synthesis (panel F), PDH (G), aconitase (H) and LDH (I) as a function of QGR for the standard non-essential amino acid (NEAA) composition of biomass NEAAA STD (reaction protein_synthesis50A50B in S1 File) and for NEAA 80–20 in which proteins are assumed to be 80% by NEAA derived from glycolysis (serine, glycine, cysteine and alanine) and for 20% by amino acids derived from glutamine (aspartate, asparagine, glutamate, glutamine, arginine, proline), and for the opposite case of NEAA 20–80 (reactions protein_synthesis80A20B and protein_synthesis20A80B, respectively, in S1 File). Note that only the rate of amino acid synthesis (e.g. serine) is affected by the change of amino acid composition in the biomass, while main carbon fluxes (pyruvate dehydrogenase, aconitase and lactate dehydrogenase) are not affected.
(PDF)

S2 Fig Similarity of results obtained when oxygen consumption is modulated by altering either oxygen availability (as in Fig 4) or Complex I capacity (flux upper bound). Note that because of the reaction stoichiometry, Complex I flux is proportionally higher than the corresponding oxygen consumption rate. A-B) Growth rate scaled on glucose availability as a function of O_2_GR (panel A) and as a function of the Complex I capacity over glucose uptake ratio (CGR in panel B) for three different levels of glutamine abundance (QGR). Purple dots highlight the growth rate when O_2_GR or CGR is optimal (oxygen not limiting for biomass). C-D) Lactate efflux scaled on glucose uptake as a function of O_2_GR (panel C) and CGR (panel D) for three different levels of QGR. Red points highlight the level of O_2_GR or CGR for which maximum lactate secretion is observed (critical OGR/CGR). E-F) Aconitase flux as a function of O_2_GR (Panel E) and CGR (Panel F). Red dots highlight the flux when O_2_GR or CGR is critical.
(PDF)

S3 Fig Interplay among the sub-networks showing alternative flux patterns in Fig 5. The first sub-network (shaded polygon, with the exception of pyruvate carboxylase) includes PEP carboxykinase; pyruvate kinase, NADP and NAD dependent malic enzyme and malate dehydrogenase. Because the TCA cycle is working in a non-cyclic mode, this set of reactions allows to maintain the steady state by removing glutamine-derived TCA cycle intermediates: OAA produced by reductive carboxylation of AKG and not used for fatty acids biosynthesis and malate deriving from fumarase, which, by means of the reactions in this subnetwork, can be converted into pyruvate and then secreted as lactate. The main options to remove OAA and malate are reported in Fig 5B–5E. Note that malate dehydrogenase and PEP carboxykinase fluxes are completely positively correlated (panel A): when PEP carboxykinase is off, malate dehydrogenase must work in the backward mode (reduction of OAA to malate). On the contrary, when malic enzyme is off and malate dehydrogenase works to oxidize malate to OAA, PEP carboxykinase must carry all the flux from TCA to pyruvate. The second sub-network (shaded oval in Fig 5) is composed by NAD and NADP IDH, which both can convert AKG to Isocitrate. As both reactions are reversible, different modes are possible to obtain the same net amount of isocitrate. Note that NAD-IDH and NADP-IDH never work in the same direction in optimal solution, as confirmed by the complete negative correlation between the two (panel B). The third sub-network (shaded rectangle in Fig 5) is composed by NADH and NADPH dependent synthesis of proline. Only two modes are possible in which the two reactions are mutually exclusive (panel C). The last sub-network is composed by NAD and NADP dependent glutamate dehydrogenase (shaded square in Fig 5). These two reactions are anti-correlated (panel D). Six different total ways to combine this pair of fluxes are consistent with optimal growth, including the two modes in which one of the two does not carry flux (as in panels B-D in Fig 5). The four networks involve redox factors and may thus influence each other. For instance, when the IDH sub-network (grey oval) works in the NADPH to NAD+ trans-hydrogenation mode, the other networks must provide the NADPH needed, as shown by the strong anti-correlation between NADP-dependent IDH and the sum of the fluxes of NADPH producing reactions: glutamate dehydrogenase and malic enzyme (panel E). Along similar lines, when the flux of NAD-IDH is in the NAD generation mode (negative flux from isocitrate to citrate), needed NADH is partially produced by GDH and malic enzyme (as suggested by the partial negative correlation between NAD-IDH and NADH production by GDH and malic enzyme in panel F).
(TIFF)

S4 Fig On why glutamine is the preferred anaplerotic precursor (supplement to Fig 6). A) Maximum and minimum value for lactate secretion across optimal growth solutions according to FVA, as a function of the parameter *pROS* that emulates the level of production of reactive oxygen species in the respiratory chain (see S1 Supplemental Methods). Glucose availability: 10; glutamine availability: 40; oxygen availability: critical level 38. B) Same experiment in panel A but with a P/O ratio of 2.5 and 1.5 considered for NADH and FADH2 respectively. C) Optimal biomass and FVA centroid of lactate secretion as a function of the ATP coefficient in the biomass forming reaction.
(PDF)

S5 Fig Determination of sample size. A) Standard deviation of the model fluxes (represented with different colors) as a function of the sample size. B) Zoom in on fluxes of panel A with low standard deviations. The fluxes with high standard deviations correspond to those with high variability when running FVA. C) Relative standard error of the mean of each flux as a function of the sample size.
(PDF)

S1 Table ENGRO1 flux distribution that maximizes growth at critical O2GR (O_2_: 38 mM h-1; G: 10 mM h-1; Q: 40 mM h-1). The optimal flux value obtained with FBA, the minimum and maximum value as well as the range size, obtained with FVA, are reported for each reaction.
(XLSX)

S2 Table ENGRO1 flux distribution that maximizes ATP production at critical O2GR (O_2_: 38 mM h-1; G: 10 mM h-1; Q: 40 mM h-1). The optimal flux value obtained with FBA, the minimum and maximum value as well as the range size, obtained with FVA, are reported for each reaction.
(XLSX)

S3 Table Analytical computations supporting simulation results in Fig 6. For different possible flux routes (table rows), the P/O ratio (column 8) of as well as the moles of acetyl-CoA deriving form one mole of substrate (column 10) following the considered route is computed. In some cases, the row indicates the difference between the computations of two possible routes (indicated as "route A rather than route B" and with a 0 for the substrate abundance value in column 1). Intermediate steps of the computation are also reported: moles of NADH (column 3) and FADH2 (column 4) obtained, moles of O_2_ consumed (column 5), ATP produced form substrate (column 6) and ATP produced by oxidation of NADH and FADH2 produced in the respiratory chain (column 7). Complex I generates ROS that may be detoxified by glutathione in a mechanism that costs one NAD(P)H per 2 molecules of superoxide anion produced. If a fraction of the electrons flowing through complex I ends up in superoxide anion, the effective P/O ratio of NADH oxidation becomes 3(1−pRos)/(1+pRos). In this computations, we assume that a mere 25% of the electrons in Complex I flow to ROS; this implies a reduction of the effective P/O for NADH from 3 to 1.8 (against a 2 P/O for FADH2). This lowers the P/O of the Gln to lactate conversion, from 3.0 to 2.2. This effect is smaller than the effect on the P/O ratio of the glucose respiration-rather-than-fermentation, i.e. from 3.0 to 2.0, explaining the preference for the flux from glutamine to lactate when so much ROS is produced by Complex I (see above). The 10% difference in P/O ratio may seem small, but as maintenance metabolism may well consume more than half the ATP produced, the difference in ATP availability for anabolism might well exceed 20%. The table considers a value of infinity (computed as 100) for the P/O ratio of the fermentation of glucose to lactate, reflecting that no oxygen is consumed in that process. When oxygen is limiting, this process comes for free and therefore glucose has a preference for this. When we compare glucose catabolism to glutamine oxidation, we correct for the phenomenon that any glucose respired comes at the cost of one glucose fermented, hence our focus on P/O ratios for glucose respiration rather than fermentation. Should lactate dehydrogenase be incapacitated, then the next best strategy is glucose respiration to CO_2_ at a P/O. Should glucose uptake be impossible but lactate dehydrogenase active, the table shows that the best flux pattern for the fittest cells is glutamine to lactate again. In none of these cases pyruvate production should be better than lactate production, but if lactate production is blocked, pyruvate production from glutamine should occur in optimal fittest cells because it is more profitable (P/O = 2.10) than complete respiration of glutamine (P/O = 2.07). These two numbers exceed the P/O of 1.82 for glucose to pyruvate rather than lactate, i.e. glucose carbon would not leave as pyruvate. The table also shows that the P/O ratio for glucose being converted to cytosolic acetyl-CoA, which serves as carbon substrate for the lipids in the biomass synthesized rather than being converted to lactate (-0.80), is more negative than that for Gln being converted to acetyl-CoA (-0.60). The clockwise pathway (at P/O = -1.8) and the combined clockwise and counterclockwise pathway for synthesis of 2 acetyl-CoA (at -1.0) from glutamine are even less favorable.
(PDF)

S1 Text Supplemental methods. Details on ENGRO1 model reconstruction.
(PDF)

S2 Text Supplemental text. Biochemical interpretation of experimental results.
(PDF)

S1 File ENGRO1 excel model. Excel file of the model compliant with the RAVEN Toolbox.
(XLS)

S2 File ENGRO1 SBML model. Excel file of the model compliant with: RAVEN Toolbox and FAME.
(XML)

S3 File ENGRO1 SVG map. Scalable Vector Graphics image of the model, compliant with the software FAME.
(SVG)

S4 File Experimental raw data. Raw data (enzyme assays, growth curves, metabolomics) used to build graphs in Fig 3.
(XLS)
