# Supplementary figures and images for: A metabolic core model elucidates how enhanced utilization of glucose and glutamine, with enhanced glutamine-dependent lactate production, promotes cancer cell growth: The WarburQ effect
Source: PLoS Comput Biol. 2017 Sep 28;13(9):e1005758. doi: 10.1371/journal.pcbi.1005758 (PMC5634631; doi:10.1371/journal.pcbi.1005758)

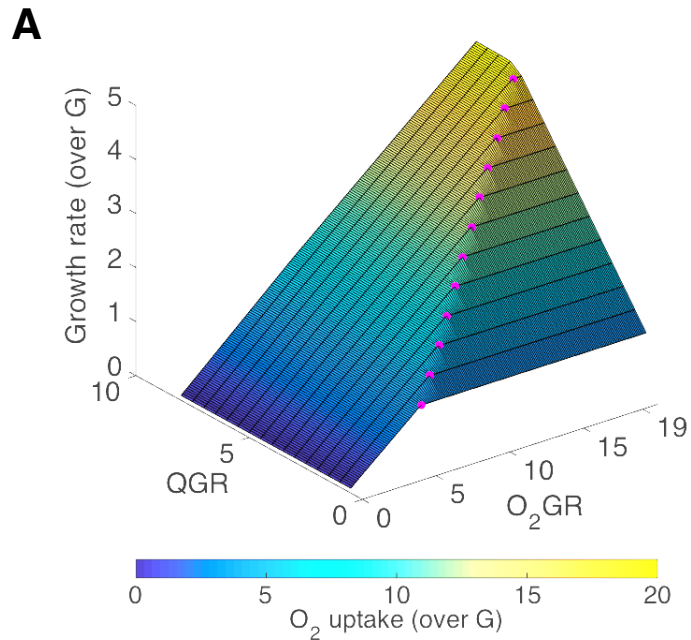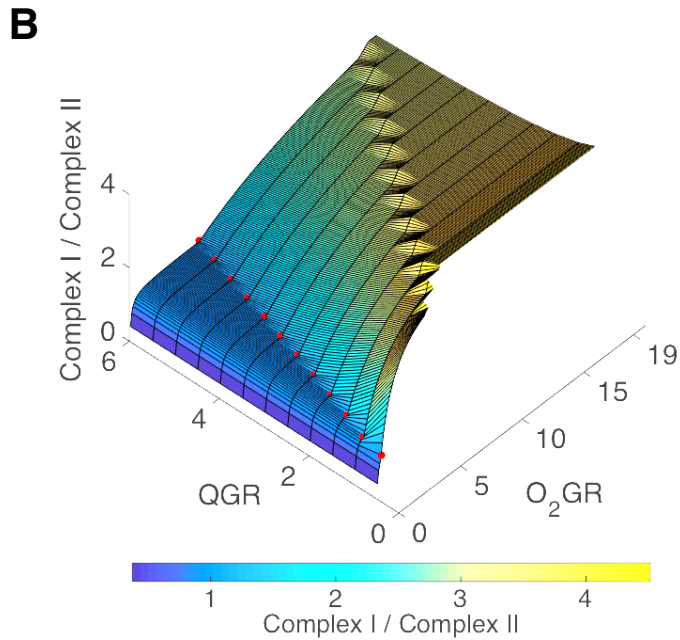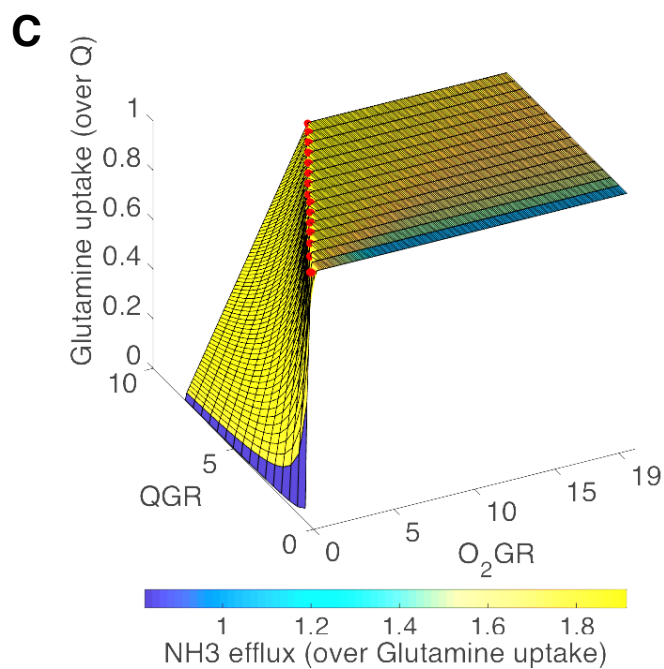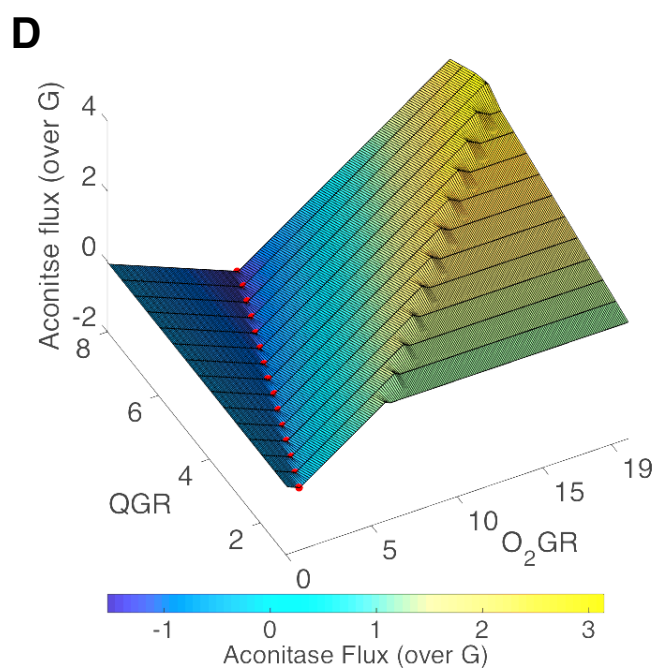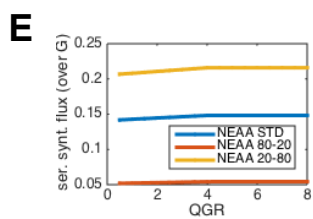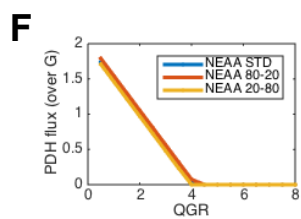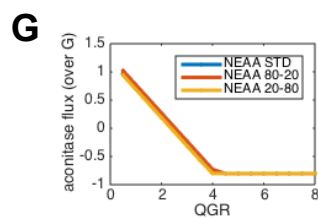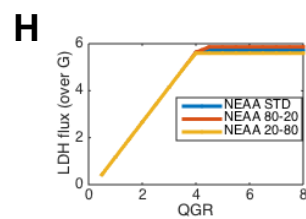

Supplement: S1 Fig — All data derive from Flux Variability Analysis in experiments that maximize biomass production. All reported fluxes do not show variability in optimal solutions. All glucose available is always fully consumed in these simulations. A) Growth rate (z-axis) and oxygen uptake (color scale) scaled on glucose uptake as a function of QGR and O2GR. The level of O2GR for which a plateau is reached (optimal O2GR) increases with the QGR (purple dots). For high O2GR a plateau is not reached within the considered range. S2 Fig show 2D slices of the 3D-mesh. B) Ratio between the flux trough Complex I and Complex II driven respiratory chain (z-axis and color scale) as a function of QGR and O2GR. Red dots highlight the value of the ratio when O2GR is critical. Full glutamine utilization takes place at Critical O2GR (panel C), when the PDH flux is 0 and the respiratory chain is fed exclusively by glutaminolysis leading to almost equal NADH (Complex I) and FADH (Complex II) oxidation, the ratio of NADH oxidation over FADH oxidation being around 1.2. C) Glutamine uptake (z-axis) and ammonia efflux (color scale)–scaled on glutamine availability and consumption respectively–as a function of QGR and O2GR. Red points highlight the minimum level of O2GR for which full glutamine consumption is observed. The increased glutamine uptake needs to be balanced by a comparable ammonia efflux that get closer to 2 when glutamine is plentiful and oxygen is low (coloring of the mesh). D) Aconitase flux (z-axis and color scale) as a function of QGR and O2GR. S2 Fig show 2D slices of the 3D-mesh. Red dots highlight the flux when O2GR is critical. At Critical O2GR, pyruvate dehydrogenase is not working, TCA cycle cannot work in a cyclic mode and production of citrate by the citrate synthase is therefore not possible. The citrate required to feed biosynthesis of fatty acids (required for biomass production) must derive from reverse carboxylation of glutamine derived AKG, as indicated by a negative flux of [file pcbi.1005758.s001.pdf]

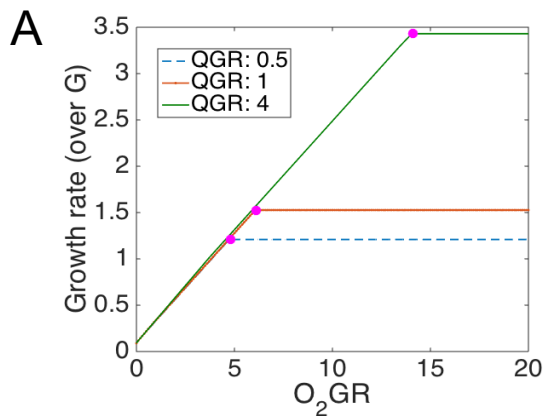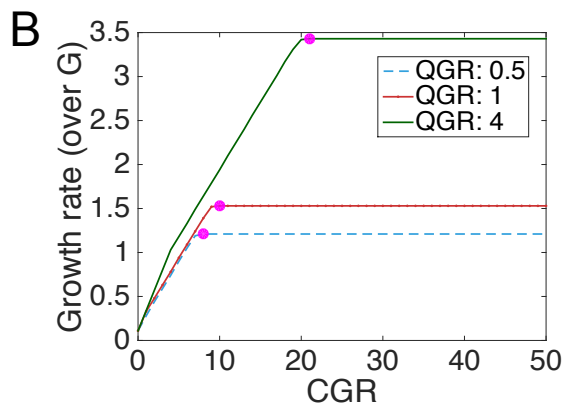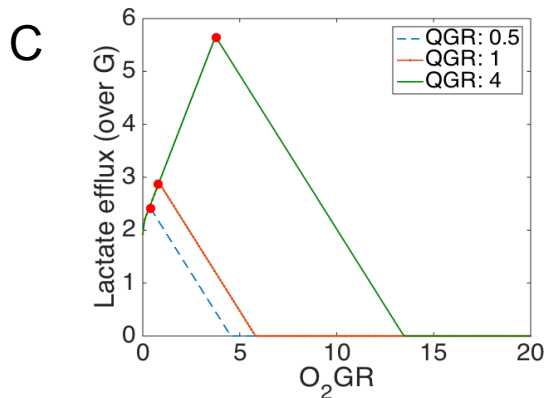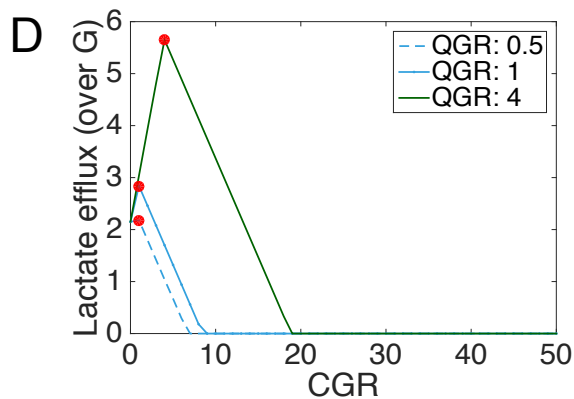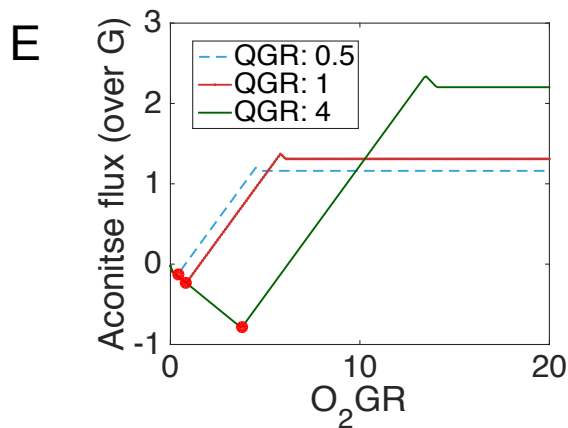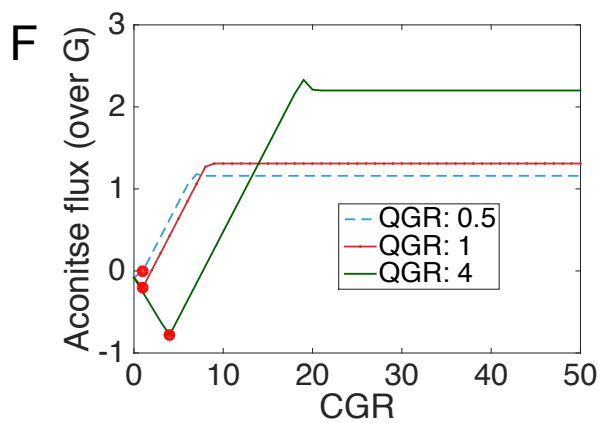

Supplement: S2 Fig — Note that because of the reaction stoichiometry, Complex I flux is proportionally higher than the corresponding oxygen consumption rate. A-B) Growth rate scaled on glucose availability as a function of O2GR (panel A) and as a function of the Complex I capacity over glucose uptake ratio (CGR in panel B) for three different levels of glutamine abundance (QGR). Purple dots highlight the growth rate when O2GR or CGR is optimal (oxygen not limiting for biomass). C-D) Lactate efflux scaled on glucose uptake as a function of O2GR (panel C) and CGR (panel D) for three different levels of QGR. Red points highlight the level of O2GR or CGR for which maximum lactate secretion is observed (critical OGR/CGR). E-F) Aconitase flux as a function of O2GR (Panel E) and CGR (Panel F). Red dots highlight the flux when O2GR or CGR is critical. (PDF) [file pcbi.1005758.s002.pdf]

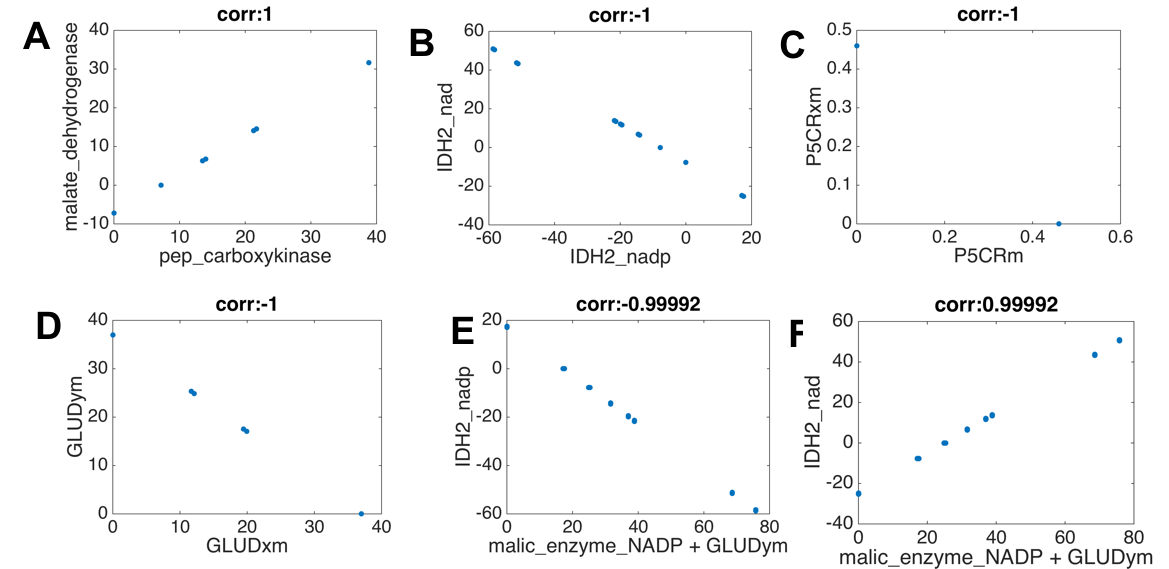

Supplement: S3 Fig — The first sub-network (shaded polygon, with the exception of pyruvate carboxylase) includes PEP carboxykinase; pyruvate kinase, NADP and NAD dependent malic enzyme and malate dehydrogenase. Because the TCA cycle is working in a non-cyclic mode, this set of reactions allows to maintain the steady state by removing glutamine-derived TCA cycle intermediates: OAA produced by reductive carboxylation of AKG and not used for fatty acids biosynthesis and malate deriving from fumarase, which, by means of the reactions in this subnetwork, can be converted into pyruvate and then secreted as lactate. The main options to remove OAA and malate are reported in Fig 5B–5E. Note that malate dehydrogenase and PEP carboxykinase fluxes are completely positively correlated (panel A): when PEP carboxykinase is off, malate dehydrogenase must work in the backward mode (reduction of OAA to malate). On the contrary, when malic enzyme is off and malate dehydrogenase works to oxidize malate to OAA, PEP carboxykinase must carry all the flux from TCA to pyruvate. The second sub-network (shaded oval in Fig 5) is composed by NAD and NADP IDH, which both can convert AKG to Isocitrate. As both reactions are reversible, different modes are possible to obtain the same net amount of isocitrate. Note that NAD-IDH and NADP-IDH never work in the same direction in optimal solution, as confirmed by the complete negative correlation between the two (panel B). The third sub-network (shaded rectangle in Fig 5) is composed by NADH and NADPH dependent synthesis of proline. Only two modes are possible in which the two reactions are mutually exclusive (panel C). The last sub-network is composed by NAD and NADP dependent glutamate dehydrogenase (shaded square in Fig 5). These two reactions are anti-correlated (panel D). Six different total ways to combine this pair of fluxes are consistent with optimal growth, including the two modes in which one of the two does not carry flux (as in panels B-D in Fig 5). The four n [file pcbi.1005758.s003.tiff]

**A**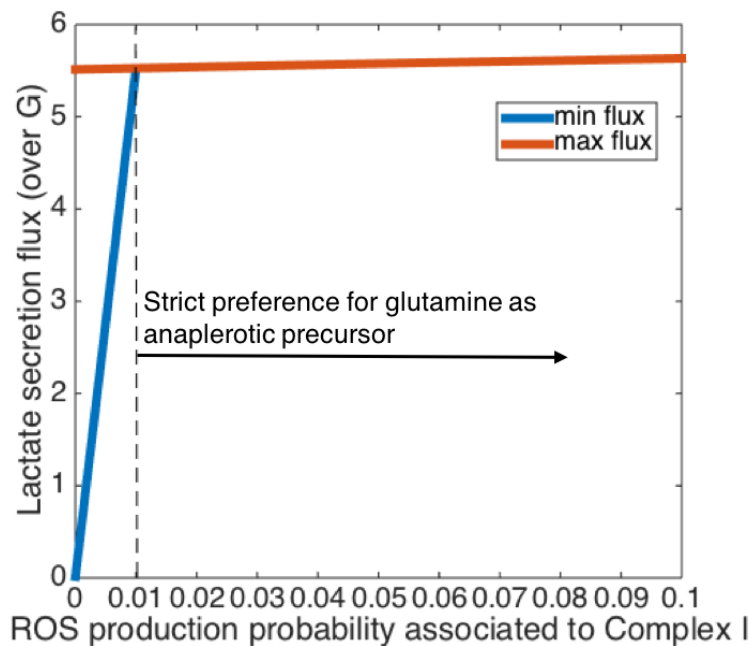**B**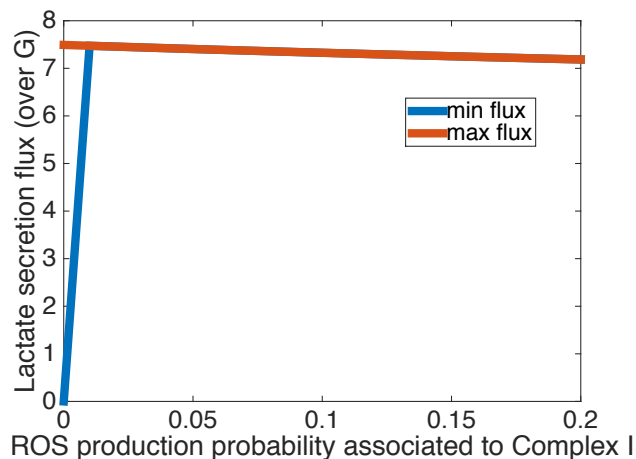**C**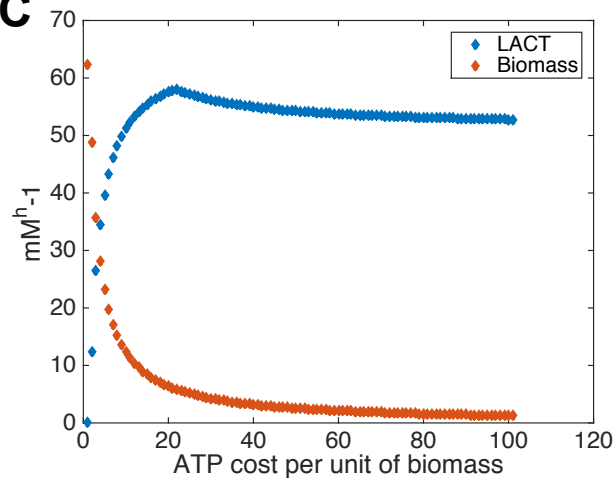

Supplement: S4 Fig — A) Maximum and minimum value for lactate secretion across optimal growth solutions according to FVA, as a function of the parameter pROS that emulates the level of production of reactive oxygen species in the respiratory chain (see S1 Supplemental Methods). Glucose availability: 10; glutamine availability: 40; oxygen availability: critical level 38. B) Same experiment in panel A but with a P/O ratio of 2.5 and 1.5 considered for NADH and FADH2 respectively. C) Optimal biomass and FVA centroid of lactate secretion as a function of the ATP coefficient in the biomass forming reaction. (PDF) [file pcbi.1005758.s004.pdf]

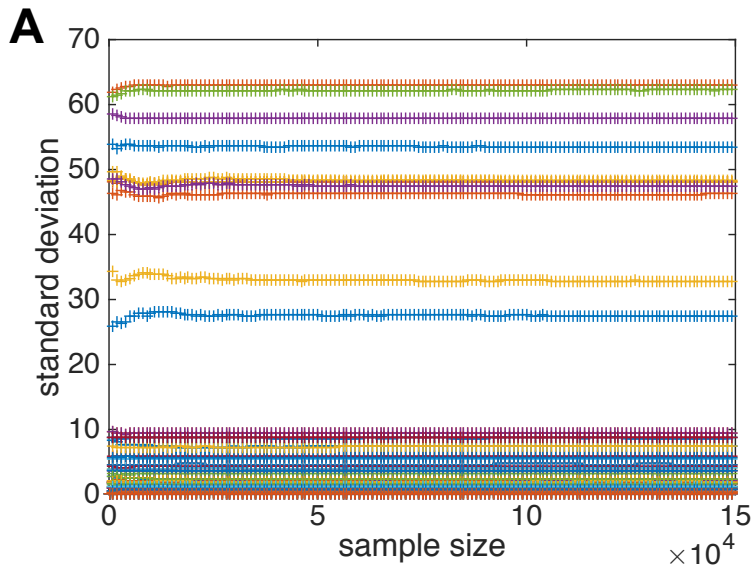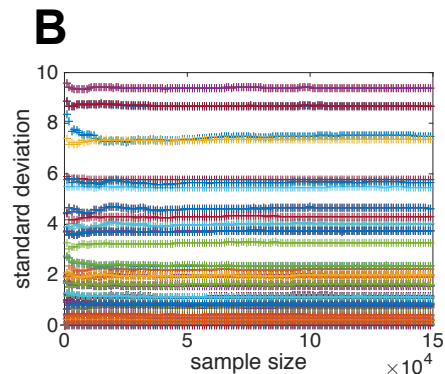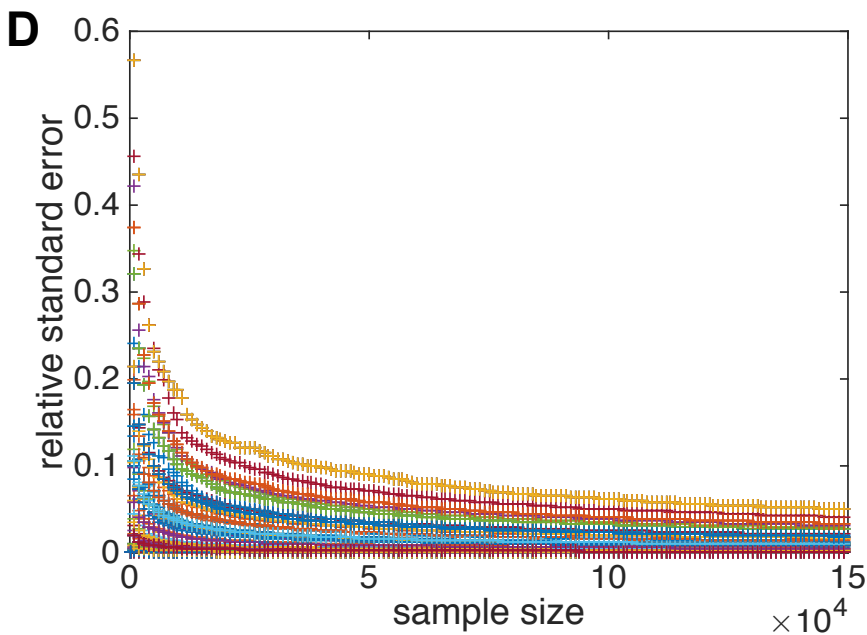

Supplement: S5 Fig — A) Standard deviation of the model fluxes (represented with different colors) as a function of the sample size. B) Zoom in on fluxes of panel A with low standard deviations. The fluxes with high standard deviations correspond to those with high variability when running FVA. C) Relative standard error of the mean of each flux as a function of the sample size. (PDF) [file pcbi.1005758.s005.pdf]
