# Supplementary material for: A metabolic core model elucidates how enhanced utilization of glucose and glutamine, with enhanced glutamine-dependent lactate production, promotes cancer cell growth: The WarburQ effect
Source: PLoS Comput Biol. 2017 Sep 28;13(9):e1005758. doi: 10.1371/journal.pcbi.1005758 (PMC5634631; doi:10.1371/journal.pcbi.1005758)
Supplement: S3 Table — For different possible flux routes (table rows), the P/O ratio (column 8) of as well as the moles of acetyl-CoA deriving form one mole of substrate (column 10) following the considered route is computed. In some cases, the row indicates the difference between the computations of two possible routes (indicated as "route A rather than route B" and with a 0 for the substrate abundance value in column 1). Intermediate steps of the computation are also reported: moles of NADH (column 3) and FADH2 (column 4) obtained, moles of O2 consumed (column 5), ATP produced form substrate (column 6) and ATP produced by oxidation of NADH and FADH2 produced in the respiratory chain (column 7). Complex I generates ROS that may be detoxified by glutathione in a mechanism that costs one NAD(P)H per 2 molecules of superoxide anion produced. If a fraction of the electrons flowing through complex I ends up in superoxide anion, the effective P/O ratio of NADH oxidation becomes 3(1−pRos)/(1+pRos). In this computations, we assume that a mere 25% of the electrons in Complex I flow to ROS; this implies a reduction of the effective P/O for NADH from 3 to 1.8 (against a 2 P/O for FADH2). This lowers the P/O of the Gln to lactate conversion, from 3.0 to 2.2. This effect is smaller than the effect on the P/O ratio of the glucose respiration-rather-than-fermentation, i.e. from 3.0 to 2.0, explaining the preference for the flux from glutamine to lactate when so much ROS is produced by Complex I (see above). The 10% difference in P/O ratio may seem small, but as maintenance metabolism may well consume more than half the ATP produced, the difference in ATP availability for anabolism might well exceed 20%. The table considers a value of infinity (computed as 100) for the P/O ratio of the fermentation of glucose to lactate, reflecting that no oxygen is consumed in that process. When oxygen is limiting, this process comes for free and therefore glucose has a preference for this. When we compare glucose cat [file pcbi.1005758.s008.pdf]

	substrate	NADH	FADH	O2	ATPsubstrate level	ATPtotal	ATPtotal per O	acetylCoA	ATP/acetylCoA	supposed ATP maintenance per O	net ATPtotal per O	percentage advantage with maintenance
**ATP production**												
glucose to 6 CO2	1.00	10.00	2.00	6.00	4.00	26.00	2.17	0.00		1.00	1.17	
glucose to lactate	1.00	0.00	0.00	0.01	2.00	2.00	100.00	0.00		1.00	99.00	
glucose to CO2 rather than lactate	0.00	10.00	2.00	6.00	2.00	24.00	2.00	0.00		1.00	**1.00**	0.0
glucose to pyruvate	1.00	2.00	0.00	1.00	2.00	5.60	2.80	0.00		1.00	1.80	
glucose to pyruvate rather than lactate	0.00	2.00	0.00	0.99	0.00	3.60	1.82	0.00		1.00	**0.82**	-18.2
gln to CO2	1.00	7.00	2.00	4.50	2.00	18.60	2.07	0.00		1.00	1.07	
gln to lactate	1.00	2.00	1.00	1.50	1.00	6.60	2.20	0.00		1.00	**1.20**	20.0
gln to CO2 rather than lactate	0.00	5.00	1.00	3.00	1.00	12.00	2.00	0.00		1.00	**1.00**	0.0
gln to pyruvate	1.00	3.00	1.00	2.00	1.00	8.40	2.10	0.00		1.00	**1.10**	10.0
**acetyl CoA production**												
glucose to acetyl CoA rather than lactate minus gln to lactate to correct for oxygen		1.33	-1.33	0.00	-1.33	-1.60		2.00	**-0.80**			
gln to acetyl CoA [c] (reductive carboxylation) and through pyruvate	1.00	0.00	0.00	0.00	-2.00	-2.00		2.00	**-1.00**			
gln to acetyl CoA [c] clockwise TCA minus gln to lactate to correct for oxygen		1.33	-1.33	0.00	-3.33	-3.60		2.00	**-1.80**			
gln to acetylCoA ccw and lactate and gln to lactate to correct for oxygen		-0.67	0.67	0.00	-1.33	-1.20		2.00	**-0.60**			
**tradeoffs**												
gln to lactate at the cost of glucose to CO2 rather than lactate							0.20					
gln to CO2 at the cost of glucose to CO2 rather than lactate							-0.80					
gln to lactate at the cost of gln to CO2							0.28					
glucose to pyruvate at the cost of glucose to lactate							-2.07					
gln to pyruvate at the cost of gln to lactate							-2.20					
gln to pyruvate at the cost of gln to CO2							0.30					
gln to acetyl CoA reductive vs glucose to acetyl CoA rather than lactate									0.20			
