# Supplementary material for: A metabolic core model elucidates how enhanced utilization of glucose and glutamine, with enhanced glutamine-dependent lactate production, promotes cancer cell growth: The WarburQ effect
Source: PLoS Comput Biol. 2017 Sep 28;13(9):e1005758. doi: 10.1371/journal.pcbi.1005758 (PMC5634631; doi:10.1371/journal.pcbi.1005758)
Supplement: S1 Text — Details on ENGRO1 model reconstruction. (PDF) [file pcbi.1005758.s009.pdf]

Supplemental Methods

ENGRO 1 model reconstruction

Pathways and metabolites included

We checked that each reaction in the model was able to carry flux. For example, we inserted a demand reaction for oxidized glutathione (GSSG) to allow steady state flux through the biosynthesis pathway for reduced glutathione (GSH) - from glycine and gamma-glutamylcysteine. Before correcting for this problem, we verified in literature that secretion of GSSG has been observed in human cells. We wanted to model this pathway because glutathione is an important antioxidant in charge of ROS removal, which may play a relevant role in cancer metabolic rewiring [1]. Notice that steady state removal of ROS would still be possible without biosynthesis of GSH, as GSH can be constantly regenerated from GSSG, by the enzyme glutathione reductase. This is the reason why, when GSH is both synthesized and regenerated, a steady state is not possible because GSSG accumulates. A demand reaction was thus inserted to remove GSSG from the network. Notice that we included also a demand reaction for reactive oxygen species (namely, H_2O_2) although we are aware that cells cannot freely dispose ROS. This choice was made to be able to assess whether cancer cells benefit from ROS removal by means of glutathione reductase, even when they are not obliged to use this pathway.

Along similar lines, release of urea cycle intermediates, such as ornithine, which is a precursor of proline, counterintuitively requires ornithine or arginine to be taken up. In fact, we did not find in biological databases the existence of anaplerotic reactions to make ornithine form precursor different from arginine, which is as well produced within the urea cycle. Because arginine is indeed typically present in cancer cells growth medium, we decided to simulate some intake of arginine.

We did not explicitly modeled the contribution of essential amino acids, exception made for methionine, for two reasons: i) if we include the catabolic pathways for the utilization of their carbon or nitrogen atoms, they might replace glucose and glutamine in central carbon metabolism, and thus we would need to systematically evaluate their consumption rates as well, resulting in combinatorial explosion of model parameters. ii) if we do not include those pathways, then the contribution of essential amino acids to growth would be trivial and depend merely upon their stoichiometry in the biomass-formation reaction. As their inclusion would not add information, we preferred to work with a more handily model, with less metabolites.

Notice that we did explicitly modeled the contribution of methionine, because it is required for the synthesis of the semi-essential amino acid cysteine, which represents a substrate of the glutathione pathway.

We explicitly modeled all non essential amino acids, exception made for the semi-essential amino acid tyrosine, which would require, as precursor, the essential amino acid phenylalanine, while not being involved in central carbon metabolism.

We avoided compartmentalization of metabolites, paying attention that this simplification would not affect the energetic requirements for biomass, based on literature and on modeling results. For example, a unique pool of acetyl-CoA would result in the capability of synthesizing fatty acids (acetyl-CoA to palmitate) directly from the acetyl-CoA produced by the pyruvate dehydrogenase mitochondrial reaction. However, it is well known that the cytosolic acetyl-CoA pool for fatty acids must derive from citrate, via the citrate lyase catalyzed reaction. Citrate in turn derives from mitochondrial acetyl-CoA, via the citrate synthase reaction (citrate and OAA to acetyl-CoA). Because the citrate lyase reaction costs one molecule of ATP, to preserve the energetic requirements of fatty acids synthesis, we did model two separate pools of acetyl-CoA (one mitochondrial and one cytosolic).

Because we are dealing with a core model, we are not taking into account all the possible pathways involving water, carbon dioxide, coenzyme A, protons and phosphates. To avoid the interplay between different pathways to rely on the competition for these metabolites in inappropriate ways, we did not explicitly model those metabolites. Notice that this simplification does not imply that our model is unbalanced (the stoichiometry of the modeled substrates is still written according to balanced reaction database), simply that the interplay among the various network pathways is not modeled for these minor metabolites, but only for the metabolites that are more comprehensively included in the network. An equivalent approximation would have been achieved if those compounds appeared in the reaction equations, while being free to be largely taken up or removed from the system.

For similar reasons, explicitly modeling of AMP and GTP molecules would result in a tight coupling

between the few reactions producing AMP or GTP and the few reactions consuming them in central carbon metabolism, unless they are let free to be uptaken/secreted. Because free uptake/release of energetic molecules would be improper, especially when dealing with biomass optimization, in order to not affect the overall energy requirements for biomass, different molecules with an equivalent amount of energy were thus considered as a unique molecular species. In order to do so, every time a GTP is used, it is substituted by ATP. Similarly, ATP-hydrolyzing reactions giving AMP and pyrophosphate were modeled as 2 ATP giving 2 ADP. This approximation allowed us to avoid unrealistic uptake of energetic molecules (which is often allowed in genome-wide models).

To further streamline the model, set of reactions belonging to a pathway with no branches were lumped into a single aggregate reaction, taking into account the net utilization of cofactors and the final products. Indeed, reactions in unbranched pathways would necessarily carry the very same flux in FBA computations. Their detail would thus not add information to simulation outcomes. By way of example: synthesis of fatty acids was modeled with a single reaction that requires 14 NADPH and 7 ATP molecules. This is the net stoichiometry of many separate reactions, which involve first consumption of 1 ATP from acetyl-CoA to malonyl-CoA. The long carbon chain of fatty acids is then assembled in a repeating sequence of four steps, two of which involve consumption of 1 NADPH molecule, that extend the fatty acyl chain by two carbons (net consumption of 2 NAPH). The cycle is repeated 7 times until the fatty acyl chain reaches 16 carbon atoms. The overall process can be thus lumped in a reaction that goes from acetyl-COA to palmitate (the first precursor to longer fatty acids), consuming a total of 7 ATP and 14 NADPH.

Along similar lines, respiratory chain is modeled as two single reactions, one that results in ATP production from NADH oxidation (via complex I, complex III, complex IV and complex V) and the other one that catalyzes ATP production from FADH2 oxidation (via complex II, complex III, complex IV and complex V).

Exchange reactions

In the model only glucose and glutamine are allowed as carbon source, while glutamine is the unique source of nitrogen.

Sink reactions that provide the network with metabolites are therefore allowed for glutamine, glucose and oxygen only. To allow mass balance, sink reactions are however included also for metabolites whose synthesis is not accounted in the network (tetrahydrofolate and methionine) and for arginine, which is required for ornithine production. Note however that the utilization of the carbon of this substrates is not allowed by ENGRO1 stoichiometry, thus their consumption will adequate to the growth capabilities of the network.

Demand reactions removing metabolites from the network are accounted for dead-end metabolites (lactate, urea, PRPP, biomass and putrescine), for GSSG (to make synthesis of GSH possible), and for H_2O_2 and NH_3.

Production of reactive oxygen species in the respiratory chain

Complex I (NADH:ubiquinone oxidoreductase) is one of the main contributors to superoxide production by mitochondria.

The reaction for the production of H_2O_2 from NADH can be written as NADH + O_2 => NAD + H_2O_2, whereas the reactions for the production of ATP from NADH oxidation according to a P/O ratio of 3 can be written as NADH + 3 ADP + 0.5 O_2 => 3 NAD + 3 ATP.

In order to impose some production of H_2O_2, we combined the two reactions by associating to NADH oxidation in the respiratory chain a probability p to produce ATP and a probability $pROS = (1 - p)$ to produce ATP according to the following equation.

$$\text{NADH} + \text{3p ADP} + \text{(1-0.5p) } O_2 \Rightarrow \text{NAD} + \text{3p ATP} + \text{(1-p) } H_2O_2$$

Parameter p takes a defult value of 0.9.

Notice that, the NADH oxidation reaction implicitly involves both complex I and complex III, so it accounts for eventual production for oxygen reactive species also by complex III.

As a first approximation, we did not associate a production of ROS to complex II, which is modeled with the FADH2 oxidation reaction (FADH2 + 0.5 O_2 + 2 ADP => FAD + 2 ATP), as ROS are predominantly produced by Complex I [3].

Definition of biomass forming equation

Biomass formation is expressed only in terms of the quantitatively most abundant macromolecular components, proteins and fatty acids, accounting for about 70% and 10% of total biomass, respectively. ENGRO1 includes the following biomass forming reaction:

0.706 protein + 0.097 palmitate + 20 ATP => biomass + 20 ADP

A protein synthesis pseudo-reaction accounts instead for the formation of proteins from the non essential aminoacids modeled in ENGRO1 following the scheme hereafter illustrated:

Ala + Glu + Gly + Pro + Asp + Cys + Ser + Gln + Asn + Arg => protein

Three different sets of stoichiometric coefficients are associated to this protein synthesis pseudo-reaction (see *protein_ synthesis*, *protein_ synthesis20A80B* and *protein_ synthesis80A20B* in S1 File).
The first set (protein_synthesis in Table ENGRO reactions list) was defined on the basis of calculations performed in [1], where the schematic fractional contribution of each amino acid in total protein and the corresponding number of molecules for each NEAA were computed starting form the relative dry weights in [4].

In order to test an unbalance between NEAA originated by glycolysis and glutamine, we recomputed the stoichiometric coefficients by splitting NEAA in the two pools proposed in [1]: a pool A including glycolytic NEAAs (serine, glycine, cysteine and alanine) and a Pool B including NEAAs from glutamine (aspartate, asparagine, glutamate, glutamine, arginine, proline).

Hence, we evaluated the two following cases where an unbalancing between the two pools is hypothesized (*protein_ synthesis20A80B* and *protein_ synthesis80A20B*):

1. proteins are constituted by NEAA deriving 80% from Pool A and 20% from Pool B
2. proteins are constituted by NEAA deriving 20% from Pool A and 80% from Pool B

We then calculated the percentage of molecules *PercA* belonging to Pool A and *PercB* belonging Pool B over the total.

In the case 1) the coefficient *Coeff* of each amminoacis were recalculated as (Coeff * 0,2) / PercA for Pool A and as (Coeff a.aB * 0,8) / PercB for Pool B. Analogous computations were performed for case 2.

Structural removal of thermodynamically infeasible cycles

Thermodynamically infeasible loops were first identified with the algorithm presented in [2] and then structurally removed by adjusting the metabolic network accordingly. In detail, two sets of reactions involved in thermodynamically infeasible loops have been identified.

1. The first set of reactions includes the glutamate gehydrogenase (GDH) reversible reactions – which utilize either NAD+ or NADP+ to catalyze the oxidative conversion of glutamate to alpha ketoglutarate – and the reversible isocitrate dehydrogenase (IDH) reactions – which utilize either NAD+ (IDH3 isoform) or NADP+ (IDH1 and IDH2 isoforms) for the oxidative decarboxylation of isocitrate, producing alpha-ketoglutarate and CO2. This loop has been adjusted by assuming both GDH reactions to be allowed exclusively in the forward reaction.
2. The second set encompasses the two IDH reversible reactions (NAD+ and NADP+ dependent) and the reversible nicotinamide nucleotide transhydrogenase (NNT) reaction – which transfers reducing equivalents from NADH to NADPH. To avoid this loop, in the first instance, the NNT reaction has been removed from the network.

Supplemental references

[1] L Alberghina and D Gaglio. Redox control of glutamine utilization in cancer. *Cell death & disease*, 5(12):e1561, 2014.

[2] Daniele De Martino, Fabrizio Capuani, Matteo Mori, Andrea De Martino, and Enzo Marinari. Counting and correcting thermodynamically infeasible flux cycles in genome-scale metabolic networks. *Metabolites*, 3(4):946–966, 2013.

[3] Michael P Murphy. How mitochondria produce reactive oxygen species. *Biochemical Journal*, 417(1):1–13, 2009.

[4] Ines Thiele, Neil Swainston, Ronan MT Fleming, Andreas Hoppe, Swagatika Sahoo, Maike K Aurich, Hulda Haraldsdottir, Monica L Mo, Ottar Rolfsson, Miranda D Stobbe, et al. A community-driven global reconstruction of human metabolism. *Nature biotechnology*, 31(5):419–425, 2013.
