# Supplementary material for: A metabolic core model elucidates how enhanced utilization of glucose and glutamine, with enhanced glutamine-dependent lactate production, promotes cancer cell growth: The WarburQ effect
Source: PLoS Comput Biol. 2017 Sep 28;13(9):e1005758. doi: 10.1371/journal.pcbi.1005758 (PMC5634631; doi:10.1371/journal.pcbi.1005758)
Supplement: S2 Text — Biochemical interpretation of experimental results. (PDF) [file pcbi.1005758.s010.pdf]

Supplemental text 2

Biochemical interpretation of experimental results

Although, FBA does not predict changes in metabolite concentrations, it can indirectly and qualitatively do so with additional information, or with simplifying assumptions such as that enzyme levels do not change.

To support interpretation of metabolic profiling results, we measured the activity of some key enzymes (Fig 3B,C), bearing in mind that when reactions involve multiple substrates and products, as enzymes can be saturated with respect to certain of their substrates, the increase of metabolite level does not necessarily lead to an increase of flux and vice versa. We assume that the reactions are not regulated in a significant way by non-substrate effectors, nor by product inhibition where higher concentration would lead to lower flux.

The model predicted that a reduction in glutaminase flux should be a consequence of a decrease in intracellular glutamine leading to a reduced intracellular glutamate, as experimentally confirmed (Fig 3D). In keeping with glutamate being the primary amino-group donor in transaminase reactions, FBA predicted a concomitant decrease in the fluxes towards most non-essential amino acids, leading to a decreased concentration of the latter, which was confirmed experimentally (Fig 3D). The essential amino acids or the amino acids that are synthesized from them without requiring a transamination step, such as threonine (Thr), methionine, cysteine and tyrosine (Tyr), should be and were confirmed to be unaltered (Fig 3D) because they are provided in the growth medium at constant concentrations (there was a similar response for ornithine that is directly derived from arginine). The flux from arginine to ornithine was predicted to be decreased, indirectly confirming the experimentally observed decrease in putrescine levels, which derives from ornithine (Fig 3D). Note that we assume that in cases of exclusively metabolic regulation the concentrations of the

intermediates of a cycle increase with flux, with few exceptions due to special kinetics of its enzymes.

This amino acid limitation should cause the predicted decreased growth rate and an increase in the ATP/ADP ratio and maintenance flux (which consumes ATP and generates ADP) and/or in the PEP-pyruvate-oxaloacetate ATP-dissipating futile cycle (Fig 3A). The increase in serine and lysine (Lys) that was experimentally observed (Fig 3D) could be a consequence of the decreased growth rate.

Our FBA computations predicted that with decreasing glutamine, the clockwise TCA cycle flux from citrate to 2-oxoglutarate should increase (Fig 3A). This increase could be achieved by gene-expression regulation, decreasing the expression levels of the mitochondrial, NADP(H)-dependent, isocitrate dehydrogenase IDH2, which is responsible for reductive carboxylation (46). This prediction was confirmed experimentally (Fig 3C). This flux change would tend to decrease the concentration of citrate, as experimentally observed (Fig 3C). The predicted decrease in the NAD(P)H/NAD(P) ratio with lowered glutamine should impede the synthesis of fatty acids (and biomass), and thereby increase the cytosolic acetyl-CoA levels, as observed experimentally (Fig 3C). This event should again increase the ATP/ADP ratio. The reduction in the succinate dehydrogenase flux predicted by FBA (Fig 3A) might be ascribed to the purported increase in the ATP/ADP ratio, because there was no expression regulation of this enzyme complex (Fig 3C) and the other substrate-product couple moved the other way.

Given that oxygen is a limiting species, the increased ATP/ADP ratio should decrease Complex II (succinate dehydrogenase) respiration flux more than Complex I flux, because the former should be expected to be more responsive to changes in the ATP/ADP ratio than the latter. In fact, Complex I produces ROS, which causes a lower effective P/O ratio so that an increased ATP/ADP ratio is less inhibitory for Complex I. Interestingly, the model indicated (Fig 3A) a strong Complex II down-regulation and moderate Complex I up-regulation. The rather strong decrease in Complex II flux predicts the experimentally observed increase in succinate, and, through the activity of fumarase,

the decrease in fumarate (Fig 3C). The decrease in fumarate and malate may have been enhanced by the predicted decrease in fumarate efflux from the urea cycle. Because the decrease in succinate dehydrogenase does not equal to that of oxygen consumption, some extra oxygen can be consumed by Complex I to fully oxidize part of the glucose. Along with an increase in Reactive Oxygen Species (ROS) formation by Complex I, FBA predicted a moderate reduction in H_2O_2 removal by the glutathione pathway; accordingly, the experimentally measured total and mitochondrial ROS levels markedly increase with decreasing glutamine (Fig 3D). In this regard, it might be interesting to consider the fact that the iron sulfur center in mitochondrial aconitase is exquisitely sensitive to superoxide anion inactivation (47). Hence, the flow of carbon and hydrogen through the TCA cycle may be directly regulated by the superoxide anion production of the electron transfer pathway through its effects on aconitase-specific activity.

With decreased glutamine, the decreased NAD(P)H should cause a decreased flow of pyruvate to lactate as observed experimentally (Fig 3B). Because lactate dehydrogenase usually functions as a near-equilibrium enzyme, the decrease in the NADH/NAD ratio should translate into a similar increase in lactate/pyruvate through the stimulation of the enzyme's flux. Lactate was determined both intra and extracellularly, and a strong increase in intracellular pyruvate with decreasing glutamine should be expected, as confirmed experimentally (Fig 3C). The decrease in NADH and the increase in the pyruvate level should also enhance the flux through the pyruvate dehydrogenase complex metabolically, because this enzyme did not change its level of activity (Fig 3C). Consequently, the flux from glycolysis should be diverted away from lactate and back towards producing mitochondrial acetyl-CoA for the TCA cycle.
